# Recent Advances in Polymerization‐Induced Self‐Assembly (PISA) Syntheses in Non‐Polar Media

**DOI:** 10.1002/anie.202308372

**Published:** 2023-07-19

**Authors:** Csilla György, Steven P. Armes

**Affiliations:** ^1^ Department of Chemistry University of Sheffield Dainton Building Sheffield South Yorkshire S3 7HF UK

**Keywords:** Diblock Copolymer, Nanoparticles, Non-Polar Media, Polymerization-Induced Self-Assembly, RAFT Polymerization

## Abstract

It is well‐known that polymerization‐induced self‐assembly (PISA) is a powerful and highly versatile technique for the rational synthesis of colloidal dispersions of diblock copolymer nanoparticles, including spheres, worms or vesicles. PISA can be conducted in water, polar solvents or non‐polar media. In principle, the latter formulations offer a wide range of potential commercial applications. However, there has been just one review focused on PISA syntheses in non‐polar media and this prior article was published in 2016. The purpose of the current review article is to summarize the various advances that have been reported since then. In particular, PISA syntheses conducted using reversible addition‐fragmentation chain‐transfer (RAFT) polymerization in various *n*‐alkanes, poly(α‐olefins), mineral oil, low‐viscosity silicone oils or supercritical CO_2_ are discussed in detail. Selected formulations exhibit thermally induced worm‐to‐sphere or vesicle‐to‐worm morphological transitions and the rheological properties of various examples of worm gels in non‐polar media are summarized. Finally, visible absorption spectroscopy and small‐angle X‐ray scattering (SAXS) enable in situ monitoring of nanoparticle formation, while small‐angle neutron scattering (SANS) can be used to examine micelle fusion/fission and chain exchange mechanisms.

## Graphical abstract

Polymerization‐induced self‐assembly (PISA) is a versatile technique for the rational synthesis of block copolymer nanoparticles in non‐polar media. In principle, such formulations offer commercial applications but only one review article has been published in this field. The current review summarizes the advances reported since 2016, including thermally induced morphological transitions, gel rheology behaviour and in situ monitoring techniques.

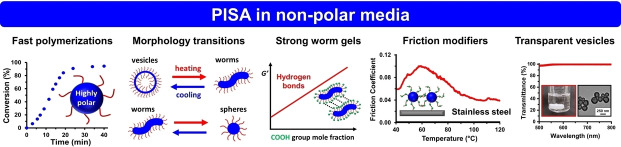

## 1 Introduction

**Figure 1:**
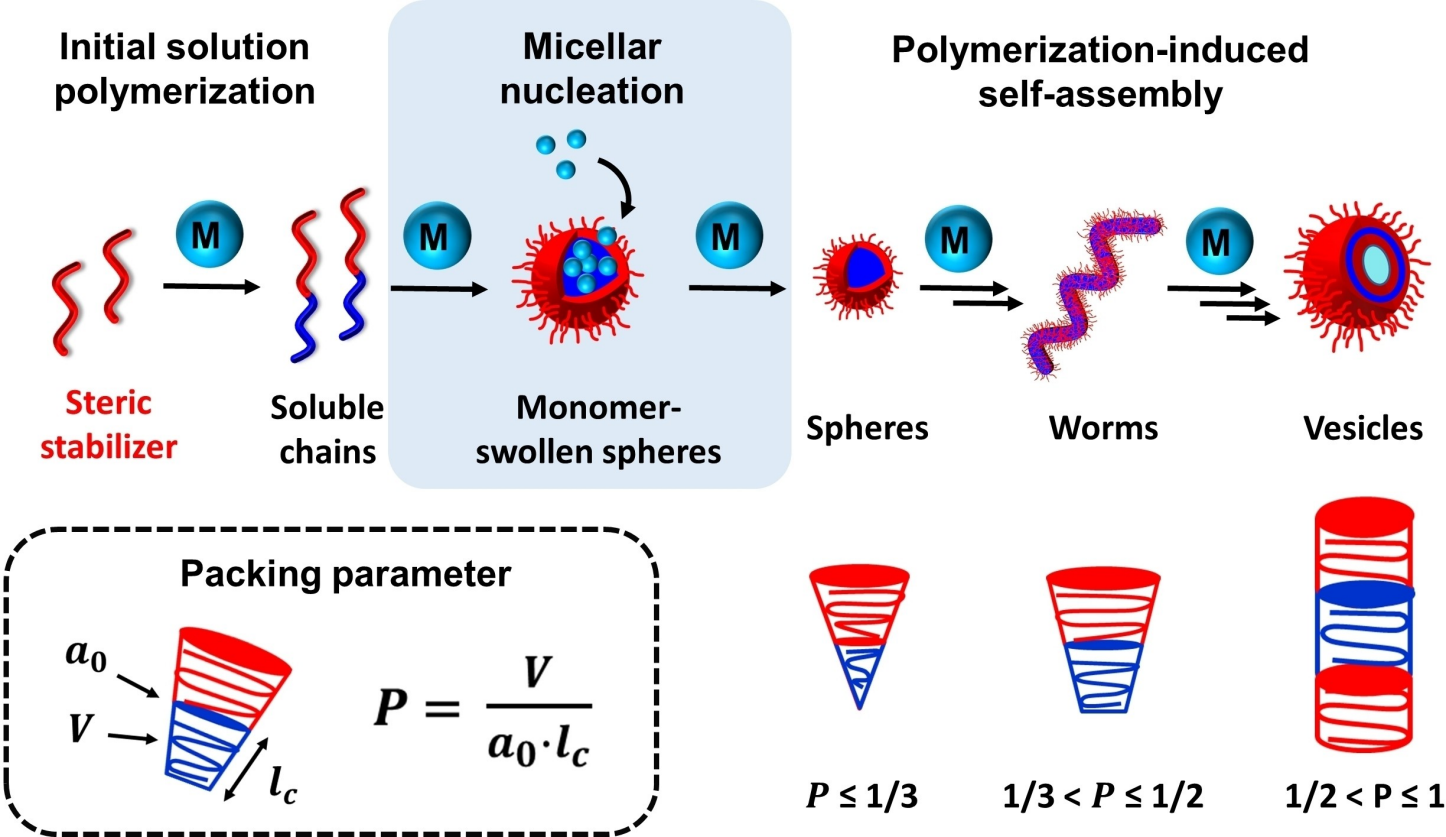
Schematic representation of the synthesis of diblock copolymer nano‐objects by polymerization‐induced self‐assembly (PISA). *V* and *l*_c_ denote the volume and the length of the core‐forming block and *a*_0_ is the effective interfacial area of the block junction.

Polymerization‐induced self‐assembly (PISA) involves growing a second block from a soluble precursor block in a suitable solvent. Initially, a solution polymerization occurs but the second block eventually becomes insoluble at some critical degree of polymerization (DP), which leads to in situ micellar nucleation. Unreacted monomer then diffuses into these nascent particles, which become the new locus for the remaining polymerization. The final product is a colloidal dispersion of sterically‐stabilized diblock copolymer nanoparticles, see Figure 1. Depending on the relative volume fraction of each block (as defined by the fractional packing parameter, *P*),[^1^, ^2^] and providing that various other conditions are also fulfilled, the final copolymer morphology can be readily adjusted to obtain spheres, worms or vesicles, see Figure 1.[^3^, ^4^, ^5^, ^6^, ^7^]

PISA is particularly attractive because it offers a highly convenient route for the efficient synthesis of diblock copolymer nano‐objects at up to 50 % w/w solids.[^3^, ^4^, ^5^, ^7^, ^8^, ^9^, ^10^, ^11^, ^12^] Moreover, the design rules for PISA are generic: this protocol works well in aqueous media, polar solvents or non‐polar media. Many PISA review articles have been published by various research groups over the past few years.[^3^, ^4^, ^5^, ^6^, ^7^, ^8^, ^10^, ^12^, ^13^, ^14^, ^15^, ^16^, ^17^] However, only one of these reviews has focused on PISA syntheses in non‐polar media (e.g., *n*‐alkanes, mineral oil, silicone oil, etc.).^[4]^ This prior article was published in 2016. The purpose of the present article is to summarize the most important advances made in this field over the past seven years. We also briefly discuss potential industrial applications for selected PISA formulations.

## 2 Discussion

### 2.1 General Remarks

There are a few examples of the use of atom transfer radical polymerization (ATRP)[^19^, ^20^, ^21^, ^22^] in either mineral oil^[23]^ or supercritical CO_2_.^[24]^ However, reversible addition‐fragmentation chain transfer (RAFT) polymerization[^25^, ^26^, ^27^, ^28^] is more typically used to conduct PISA syntheses in non‐polar media. RAFT polymerization utilizes an organosulfur compound such as a trithiocarbonate or a dithiobenzoate. Such chain transfer agents react rapidly and reversibly with vinyl polymer radicals, which are usually generated via thermal decomposition of a conventional azo or peroxide initiator. This reduces the probability of termination and leads to so‐called pseudo‐living polymerizations that exhibit a linear evolution in molecular weight with monomer conversion, a relatively narrow molecular weight distribution, and the formation of well‐defined diblock copolymer chains on addition of a second vinyl monomer after consumption of the first vinyl monomer. Moreover, the radical nature of a RAFT polymerization means that it is highly tolerant of monomer functionality. However, intrinsic disadvantages of such chemistry are that the organosulfur RAFT agent confers color (typically yellow or pink) and is usually rather malodorous. Removal of such end‐groups from the copolymer chains can be achieved by post‐polymerization modification using various chemistries.[^29^, ^30^] However, it is rarely cost‐effective to do so for many potential applications. Nevertheless, RAFT PISA syntheses in non‐polar media have been examined by companies such as Lubrizol, L'Oréal and Ashland to produce nanoparticles for lubrication applications[^18^, ^31^, ^32^] or personal care products.[^33^, ^34^]

In principle, diblock copolymer nanoparticles can be prepared in non‐polar media via RAFT PISA using either a two‐pot[^33^, ^35^, ^36^, ^37^] or a one‐pot protocol.[^37^, ^38^, ^39^, ^40^] In the former approach, the first block is synthesized via RAFT solution polymerization, isolated and purified, then chain‐extended in the desired non‐polar solvent. Alternatively, the one‐pot protocol involves using the same non‐polar solvent for both the synthesis of the first block and its subsequent chain extension. In this case, no purification of the oil‐soluble precursor is undertaken. Typically, synthesis of the second block involves RAFT dispersion polymerization because the vinyl monomer is usually miscible with the non‐polar solvent.

**Figure 2:**
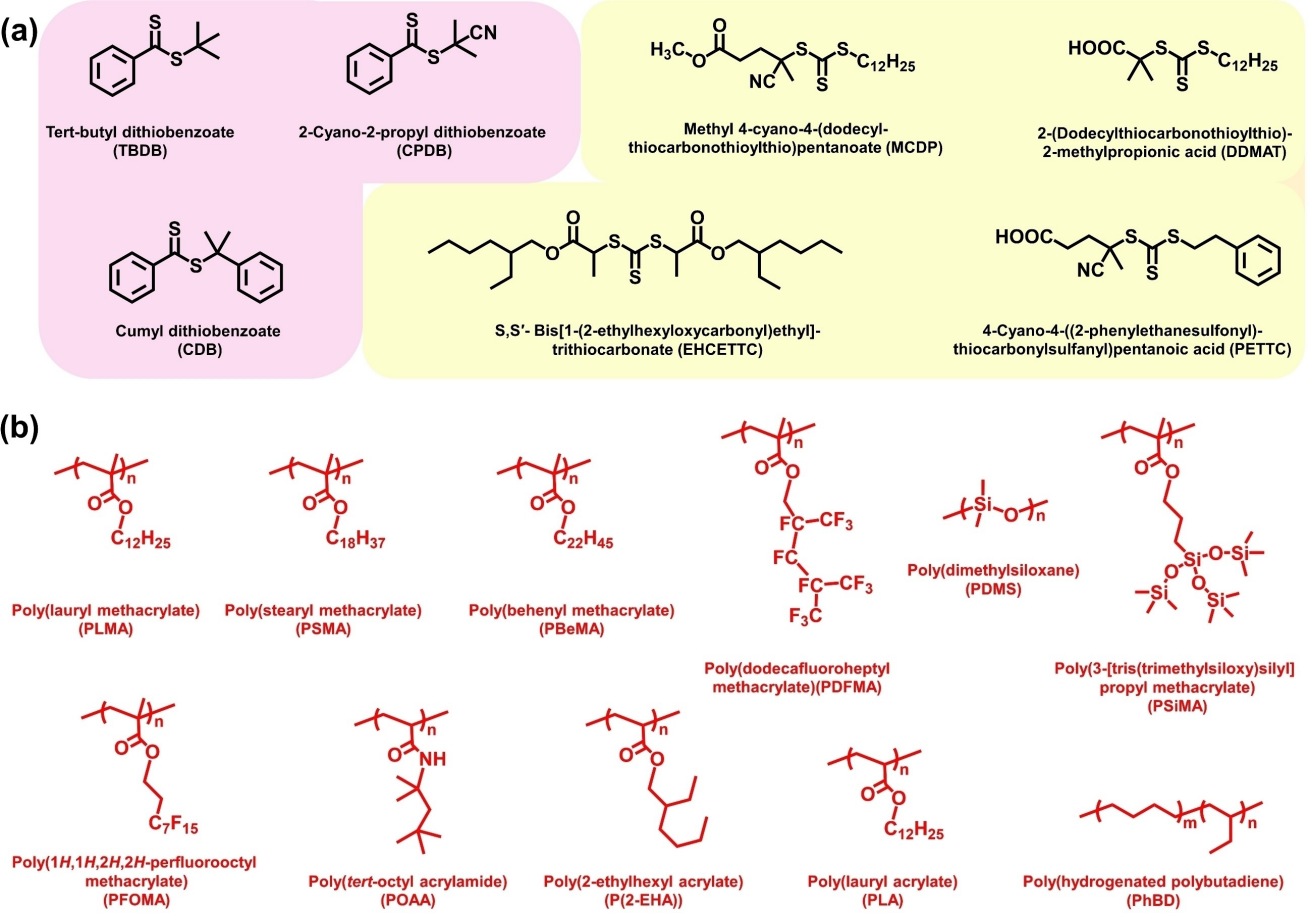
(a). Chemical structures for dithiobenzoate‐based (pink highlighted) and trithiocarbonate‐based (yellow highlighted) chain transfer agents (CTAs) used for RAFT PISA syntheses in non‐polar media. (b) Summary of the chemical structures of the various polymers employed as the steric stabilizer for RAFT PISA syntheses in non‐polar media.

**Figure 3:**
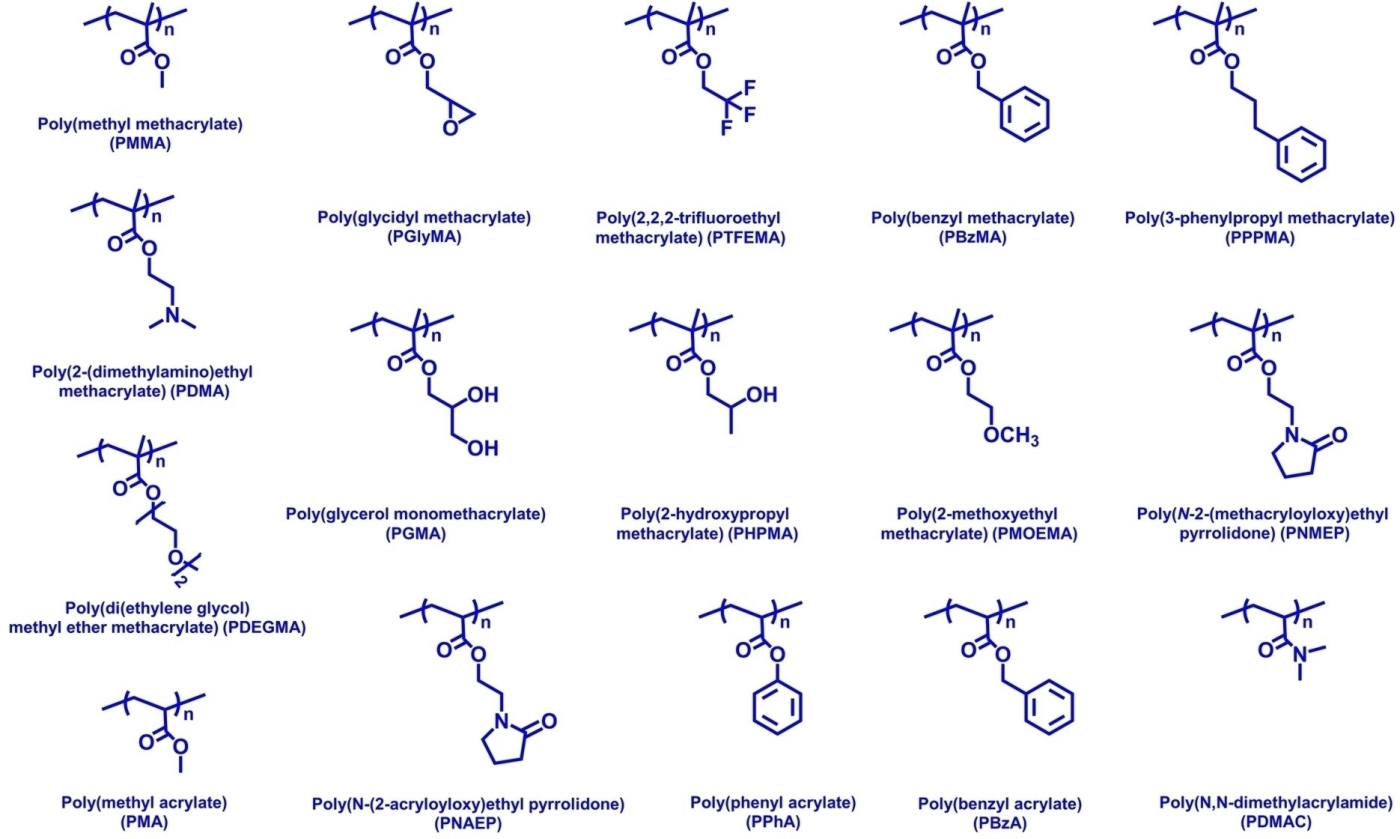
Summary of the chemical structures of the various vinyl polymers employed as the core‐forming block for RAFT PISA syntheses in non‐polar media.

Various RAFT chain transfer agents (CTAs) have been employed for preparing a wide range of diblock copolymer nano‐objects in non‐polar media, including dithiobenzoates[^35^, ^36^, ^37^, ^41^, ^42^] (e.g., CPDB or CDB, see Figure 2a) and trithiocarbonates,[^33^, ^35^, ^39^, ^43^, ^44^, ^45^, ^46^] (e.g., DDMAT, PETTC or MCDP, see Figure 2a). Similarly, various acrylic,[^33^, ^35^, ^38^, ^47^] methacrylic[^31^, ^36^, ^37^, ^42^] or acrylamide monomers^[39]^ have been utilized to produce the steric stabilizer (see Figure 2b) and core‐forming blocks (see Figure 3). In addition, monohydroxyl‐functional precursors such as polydimethylsiloxane or hydrogenated polybutadiene have been converted into macromolecular RAFT agents to serve as steric stabilizers.[^48^, ^49^, ^50^] A series of alkanes [e.g., *n*‐heptane,[^36^, ^38^, ^39^, ^47^, ^51^] *n*‐octane,[^39^, ^52^] *n*‐decane,^[39]^ *n*‐dodecane,[^44^, ^46^] *n*‐tetradecane,[^43^, ^53^] *n*‐hexadecane,^[39]^ *iso*‐dodecane[^33^, ^35^] or *iso*‐hexadecane^[38]^] have been employed as solvents, as well as poly(α‐olefins) (PAO),^[37]^ mineral oil,[^31^, ^37^, ^42^] low‐viscosity silicone oils[^49^, ^50^, ^54^] or supercritical CO_2_.[^55^, ^56^]

A typical example of a suitable core‐forming monomer is benzyl methacrylate (BzMA). The kinetics of RAFT dispersion polymerization of this monomer has been extensively studied in various solvents using either poly(lauryl methacrylate) (PLMA) or poly(stearyl methacrylate) (PSMA) as a steric stabilizer block.[^36^, ^42^] The initial solution polymerization proceeds relatively slowly, but a significant rate enhancement occurs after micellar nucleation owing to the relatively high local concentration of BzMA within the nascent monomer‐swollen nanoparticles (see Figure 1). Subsequently, a slower rate of polymerization is observed towards the end of the reaction under monomer‐starved conditions. In most cases, 99 % BzMA conversion can be achieved within 5 h at elevated temperature but the precise kinetics depends on the target diblock copolymer composition and the reaction conditions (e.g., monomer concentration, initiator type, temperature, solids content etc.).

### 2.2 Methacrylic PISA Formulations with Non‐Polar Monomers

**Figure 4:**
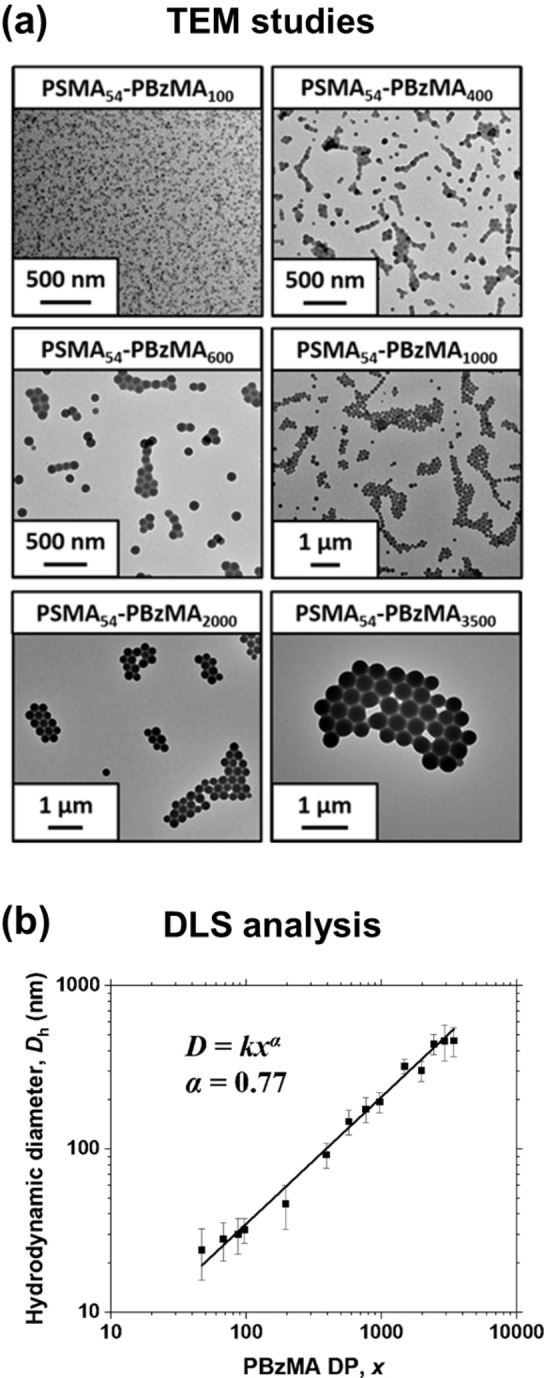
(a) Representative TEM images recorded for selected PSMA_54_‐PBzMA_x_ spherical nanoparticles prepared by RAFT dispersion polymerization of BzMA in mineral oil at 90 °C. (b) Hydrodynamic DLS diameter (*D*_h_) vs. PBzMA DP (x) obtained for a series of PSMA_54_‐PBzMA_x_ spheres (*x*=50–3500) prepared by RAFT dispersion polymerization of BzMA in mineral oil at 90 °C when targeting 20 % w/w solids. Error bars represent the standard deviation in *D*_h_ as calculated from the DLS polydispersity index. Figure adapted from Ref. [45] with permission.

Parker et al. examined a PSMA‐PBzMA formulation to establish the upper size limit for sterically‐stabilized spherical nanoparticles in mineral oil.^[45]^ Employing a relatively long PSMA_54_ precursor led to the formation of well‐defined, kinetically‐trapped spherical nanoparticles of up to 459 nm diameter at 20 % w/w solids when targeting PBzMA DPs up to 3500 (see Figure 4).

These are amongst the largest spherical nanoparticles prepared via RAFT‐mediated PISA in non‐polar media. A double‐logarithmic plot indicated a linear evolution in DLS diameter when targeting PBzMA DP of up to 2500 (see Figure 4b). However, deviation from this linear relationship was observed when targeting higher DPs, which also produced nanoparticles with relatively broad size distributions. Moreover, RAFT control was gradually lost when targeting higher PBzMA DPs, with *Đ* increasing from 1.14 (DP=50) up to 3.41 (DP=3500). Thus one important conclusion from this study is that highly polydisperse copolymer chains can form near‐mondisperse particles.

Pei et al. reported the post‐polymerization modification of nanoparticles in *n*‐octane and *n*‐tetradecane.^[53]^ First, an oil‐soluble steric stabilizer was produced via statistical copolymerization of highly reactive pentafluorophenyl methacrylate (PFPMA) with SMA. This precursor was then used to prepare poly(phenylpropyl methacrylate) (PPPMA)‐core spheres, worms or vesicles. Such (P(SMA_36_‐*stat*‐PFPMA_2_)‐PPPMA_79_) spherical nanoparticles were then modified via nucleophilic acyl substitution of the PFPMA block using benzylamine, *N,N*‐dimethylethylenediamine, tetrahydrofurfuryl amine, or methyl red amine in the presence of *n*‐butyl acrylate, which acted as a Michael acceptor. This approach generated a library of functional nanoparticles.

**Figure 5:**
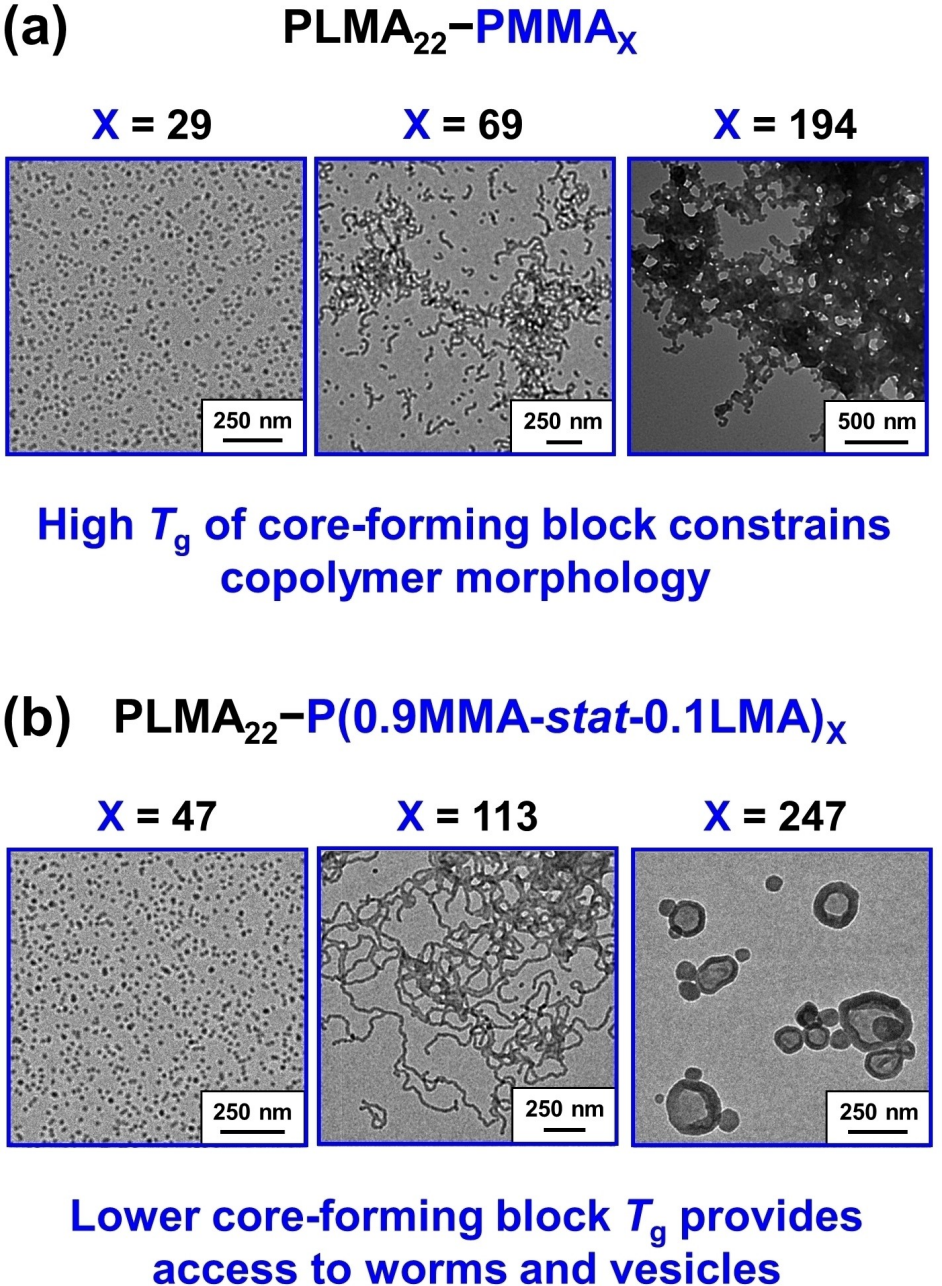
Representative TEM images recorded for (a) PLMA_22_‐PMMA_29_ spheres, short PLMA_22_‐PMMA_69_ worms and colloidally unstable aggregates of PLMA_22_‐PMMA_194_ spheres and (b) PLMA_22_‐P(0.9MMA‐0.1LMA)_47_ spheres, PLMA_22_‐P(0.9MMA‐0.1LMA)_113_ worms and PLMA_22_‐P(0.9MMA‐0.1LMA)_247_ vesicles prepared at 20 % w/w solids in mineral oil at 90 °C. Figure adapted from Refs. 40, 59 with permission.

Obtaining higher order morphologies can often be challenging for certain PISA formulations.[^57^, ^58^] For example, György et al. reported that relatively long worms or vesicles could not be accessed when using a PLMA‐PMMA PISA formulation in mineral oil.^[40]^ Even a relatively short PLMA_22_ stabilizer block only provided access to PLMA_22_‐PMMA_x_ spheres (*x*=19–39) and relatively short worms (*x*=69–97), while targeting higher×values (x≥108) invariably led to colloidally unstable aggregates of spheres (see Figure 5a). This unexpected morphological constraint was attributed to the relatively high glass transition temperature (*T*_g_) of the core‐forming PMMA block. This problem could not be overcome by targeting higher solids, choosing a different solvent (e.g., *n*‐dodecane instead of mineral oil), or by employing an alternative steric stabilizer block (PSMA). Even raising the synthesis temperature from 90 to 115 °C (i.e., above the *T*_g_ of the final PMMA block) did not alleviate this problem. In a follow‐up study, György et al. found that incorporating 10 mol% LMA into the core‐forming block via statistical copolymerization of MMA with LMA at 115 °C provided convenient access to spheres, worms and vesicles when using the same PLMA_22_ stabilizer block (see Figure 5b).^[59]^ Several research groups have demonstrated that introducing a small amount of a solvophilic monomer into the nanoparticle core enhances plasticization of the growing insoluble chains, which results in a higher packing parameter and hence provides access to worms or vesicles.[^60^, ^61^, ^62^] In this case, the relatively low *T*_g_ of PLMA is important because introducing LMA comonomer led to a significant reduction in the *T*_g_ of the core‐forming block (e.g., PLMA_22_‐PMMA_192_, core block *T*_g_=111 °C; PLMA_22_‐P(0.9MMA‐*stat*‐0.1LMA)_188_, core block *T*_g_=82 °C). Moreover, relatively short PLMA_22_‐PMMA_69_ worms exhibited only a partially reversible worm‐to‐sphere transition at 20 % w/w solids on heating from 20 °C to 150 °C.^[40]^ In contrast, relatively long PLMA_22_‐P(0.9MMA‐*stat*‐0.1LMA)_113_ worms exhibited a fully reversible morphological transition^[59]^ similar to that reported for the PLMA_16_‐PBzMA_37_ worms discussed above.^[63]^

Häkkinen et al. reported the PISA synthesis of PLMA‐PBzMA graft copolymers by grafting from a PLMA backbone during the RAFT dispersion polymerization of BzMA in n‐dodecane.^[64]^ In this study, two critical structural parameters were identified: (i) the backbone concentration, which influenced the degree of entanglements and thus whether macroscopic gelation occurred, and (ii) the targeted graft length, which determined the core morphology. SAXS analysis revealed that increasing the mean graft DP from 1 to 105 when using a PLMA_915_‐CTA_10%_ backbone provided access to multicore micelles, worm‐like particles, vesicles and inverted multicore micelles. The latter morphology was attributed to the physical constraints imposed by the branched copolymer architecture. Furthermore, the authors proposed that the morphology evolved during the BzMA polymerization from individual copolymer chains to oblate ellipsoids to inverted multicore micelles.

**Figure 6:**
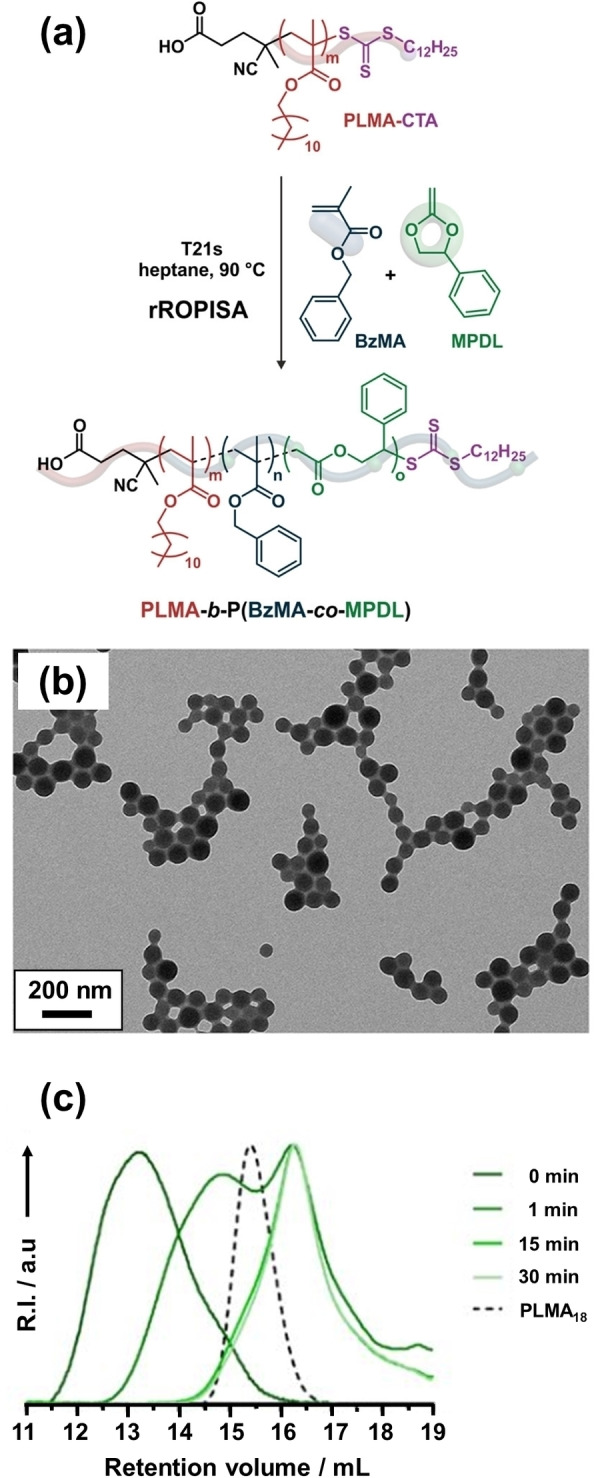
(a) Radical ring‐opening copolymerization‐induced self‐assembly (rROPISA) of BzMA and MPDL for the synthesis of degradable diblock copolymer nanoparticles. (b) Representative TEM image obtained when targeting PLMA_18_‐P(0.6BzMA‐*co*‐0.4MPDL)_250_ nanoparticles at 15 % w/w solids in *n*‐heptane. (c) Chloroform GPC data recorded for the same diblock copolymer during its degradation under accelerated conditions (2.5 % KOH in THF/methanol). The dashed line represents the GPC trace recorded for the PLMA_18_ precursor. Figure adapted from Ref. [51] with permission.

Interestingly, Guégain et al. used radical ring‐opening copolymerization‐induced self‐assembly (rROPISA) to introduce cyclic ketene acetals (CKAs) such as 2‐methylene‐4‐phenyl‐1,3‐dioxolane (MPDL) or 5,6‐benzo‐2‐methylene‐1,3‐dioxepane (BMDO) into the core‐forming block for a PLMA‐PBzMA formulation (see Figure 6a).^[51]^ Such PISA syntheses were conducted in *n*‐heptane at 90 °C and the CKA comonomer content was systematically varied. Well‐defined spherical nanoparticles were produced with mean diameters ranging from 40 to 500 nm, as judged by DLS and TEM analysis (see Figure 6b). However, increasing the CKA content led to broader nanoparticle size distributions and incomplete conversions (58–93 %). Moreover, a significant proportion of the CKA comonomer remained unreacted when targeting higher CKA contents. Nevertheless, such nanoparticles were shown to be susceptible to hydrolytic degradation owing to the backbone ester groups introduced by the MPDL or BMDO comonomers (see Figure 6a). Copolymer degradation was assessed under accelerated conditions (2.5 % KOH in THF/methanol mixtures) using chloroform GPC. A systematic shift towards lower *M*_n_ was observed (see Figure 6c) and higher CKA contents led to greater extents of degradation. However, it is perhaps questionable whether such nanoparticles could exhibit a commercially useful degradation profile given that water is immiscible with *n*‐heptane.

Supercritical CO_2_ (scCO_2_) is a sustainable non‐polar solvent for PISA syntheses that enables the solvent density and dielectric constant to be tuned by simply adjusting the temperature or pressure.^[56]^ In principle, if a suitable steric stabilizer can be identified that is soluble in scCO_2_, then either BzMA or MMA can be used for dispersion polymerization formulations.[^55^, ^56^]

**Figure 7:**
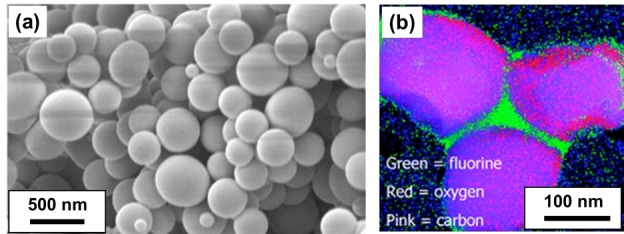
(a) SEM image of polydisperse PMMA particles prepared by RAFT dispersion polymerization in scCO_2_ using a PFOMA precursor. (b) TEM‐EDX image and elemental map confirming the presence of this perfluorinated steric stabilizer block at the surface of such particles (pink=carbon, red=oxygen, and green=fluorine). Figure adapted from Ref. [55] with permission.

In 2008, Zong et al. reported that a fluorinated poly(1*H*,1*H*,2*H*,2H‐perfluorooctyl methacrylate) (PFOMA) precursor can be employed as a steric stabilizer block for the RAFT dispersion polymerization of MMA.^[55]^ Good control over the polymerization was confirmed by THF GPC analysis and the kinetics of polymerization was monitored via ^1^H NMR spectroscopy. Scanning electron microscopy (SEM) studies indicated a well‐defined spherical morphology (see Figure 7a). Furthermore, transmission electron microscopy with energy‐dispersive X‐ray analysis (TEM‐EDX) was used to provide elemental maps of the cross‐sectioned particles. This technique indicated a well‐defined halo of fluorine atoms surrounding each spherical particle, which indicated that the perfluorinated PFOMA acted as an effective steric stabilizer in such PISA syntheses (see Figure 7b).

**Figure 8:**
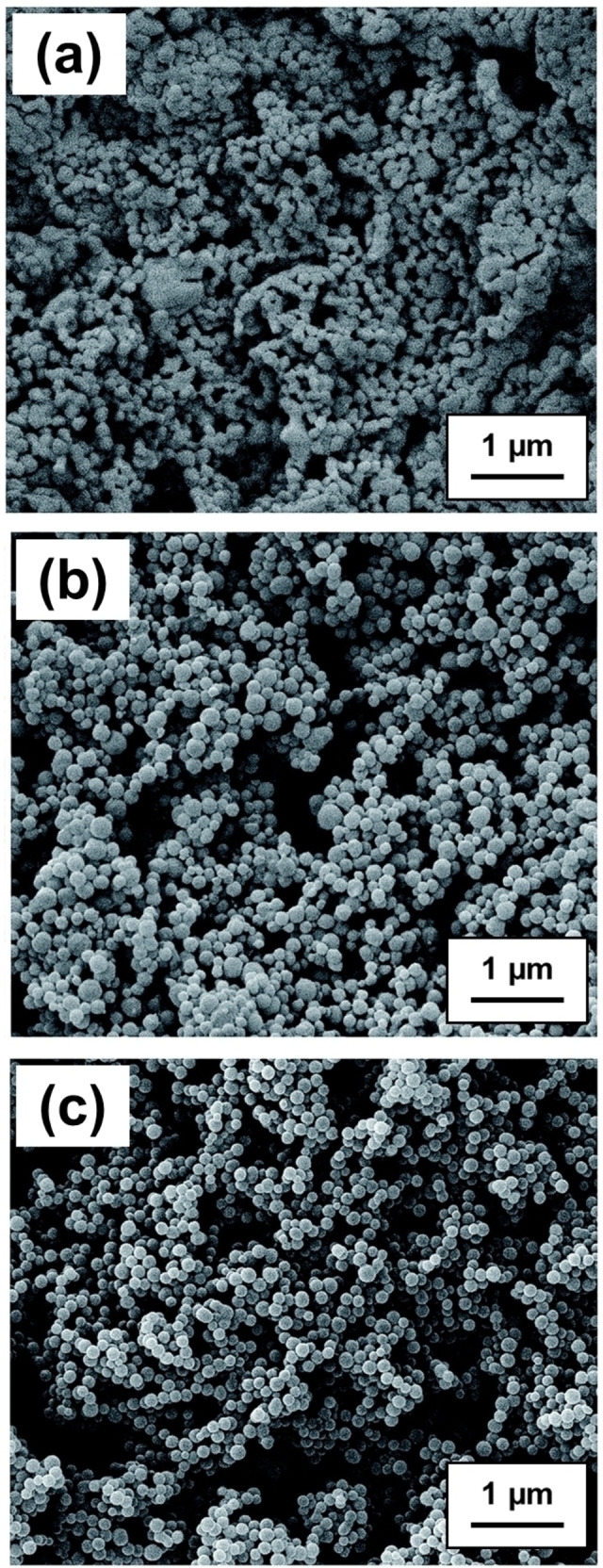
SEM images of PDFMA‐PMMA block nanoparticles prepared in scCO_2_ at various pressures. Polymerizations performed using [MMA]=0.8 M, MMA/PDFMA_32_‐CDB/AIBN=692/1/0.5 (mol % in feed) at 70 °C for 24 h at (a) 10 MPa, (b) 20 MPa and (c) 30 MPa, respectively. Figure adapted from Ref. [56] with permission.

Similarly, Xu et al. utilized an alternative fluorinated precursor, poly(dodecafluoroheptyl methacrylate) (PDFMA) to produce PDFMA_x_‐PMMA_y_ spherical nanoparticles via RAFT PISA in scCO_2_.^[56]^ In this case, the effect of varying the DPs for the steric stabilizer and the core‐forming blocks on the nanoparticle size was examined. To produce well‐defined spherical nanoparticles, the core‐forming PMMA block DP had to be at least 500 when using a relatively short PDFMA steric stabilizer DP of 15. Increasing the PDFMA DP from 15 to 55 when targeting the same PMMA DP of 500 resulted in a significant reduction in the mean nanoparticle diameter from 259 to 81 nm and narrower particle size distributions were also obtained. Furthermore, increasing the CO_2_ pressure for such formulations only led to a minimal change in the nanoparticle diameter (from 141 nm at 10 MPa to 153 nm to 30 MPa). On the other hand, SEM analysis provided evidence for nanoparticle agglomeration at lower pressures (see Figure 8), while significantly narrower particle size distributions were obtained at 30 MPa. This was attributed to the greater solubility of the PDFMA stabilizer chains in scCO_2_ under the latter conditions.

Recently, Alzaharani et al. used ATRP to produce PDMS‐PBzMA nanoparticles via PISA in scCO_2_.^[24]^ The PDMS‐Br (*M*_n_=5,350 g mol^−1^; *M*_w_/*M*_n_=1.12) precursor was obtained by reacting commercial monohydroxyl‐functionalized PDMS with 2‐bromoisobutyryl bromide. This precursor was then used to grow PBzMA blocks with target DPs of up to 400. TEM studies indicated that worms (and possibly vesicles) could be accessed in addition to spheres. Moreover, more complex morphologies could be obtained in some cases. According to the authors, this suggests that dispersion polymerization in scCO_2_ may differ from PISA conducted in other non‐polar solvents.

### 2.3 Nanoparticle Cores Comprising Polar Monomers

**Figure 9:**
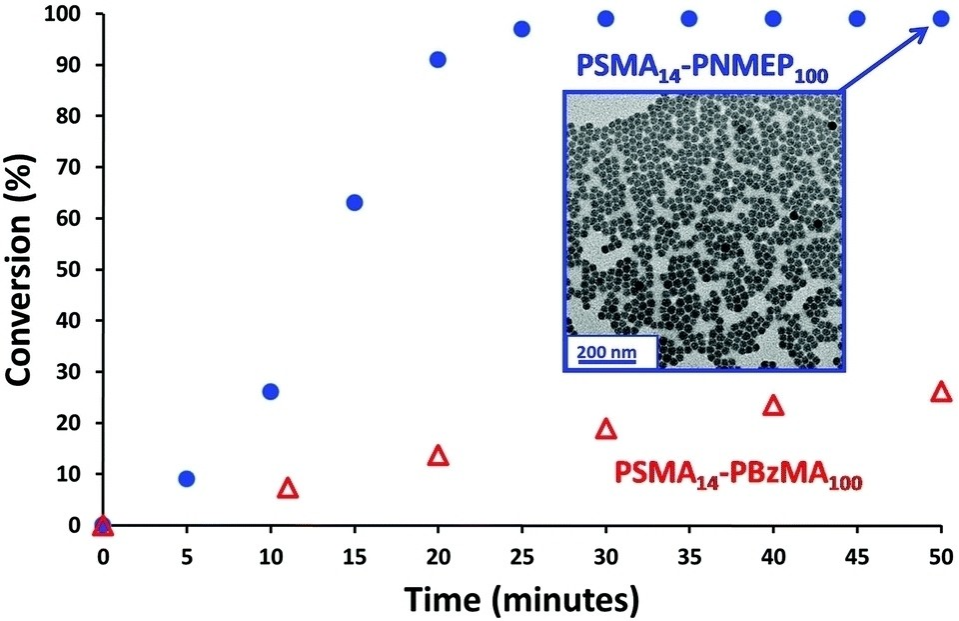
Kinetics of polymerization obtained during the RAFT dispersion polymerization of either NMEP or BzMA at 90 °C when targeting either PSMA_14_‐PNMEP_100_ (blue circles) or PSMA_14_‐PBzMA_100_ (red triangles) at 20 % w/w solids. Insert: transmission electron microscopy image obtained after 50 min (>99 % NMEP conversion) for PSMA_14_‐PNMEP_100_ nanoparticles indicating a well‐defined spherical morphology with a mean number‐average diameter of 27 nm. Figure adapted from Ref. [65] with permission.

In addition to non‐polar monomers such as PPPMA, BzMA and MMA, highly polar monomers can also be used to form the core‐forming block when conducting PISA syntheses in non‐polar media.[^31^, ^41^, ^65^, ^66^, ^67^, ^68^] Such formulations typically lead to much faster rates of polymerization. For example, Cunningham and co‐workers undertook a kinetic study of the RAFT dispersion polymerization of *N*‐2‐(methacryloyloxy)ethyl pyrrolidone) (NMEP) in *n*‐dodecane using ^1^H NMR spectroscopy.^[65]^ An NMEP conversion of≥99 % was achieved within 30 min when using a PSMA_14_ steric stabiliser to target a PNMEP DP of 100 at 20 % w/w solids. In contrast, less than 20 % benzyl methacrylate (BzMA) conversion was obtained within the same time scale for the corresponding PSMA_14_‐PBzMA_100_ synthesis conducted under the same reaction conditions (see Figure 9).

Similarly, György et al. compared the kinetics of RAFT dispersion polymerization of 2‐hydroxypropyl methacrylate (HPMA) with that of BzMA in mineral oil using a PSMA_9_ precursor.^[66]^ In this case, the polymerization kinetics was studied during the synthesis of PSMA_9_‐PHPMA_150_ vesicles and PSMA_9_‐PBzMA_150_ vesicles in mineral oil when targeting either 15 % w/w or 18 % w/w solids (i.e., [HPMA]_0_=[BzMA]_0_=0.78 M), respectively. An HPMA conversion of 94 % was observed within 40 min, whereas only 37 % BzMA conversion was obtained on the same timescale. As far as we are aware, this is the only PISA formulation that has led to the formation of block copolymer lamellae in non‐polar media.

**Figure 10:**
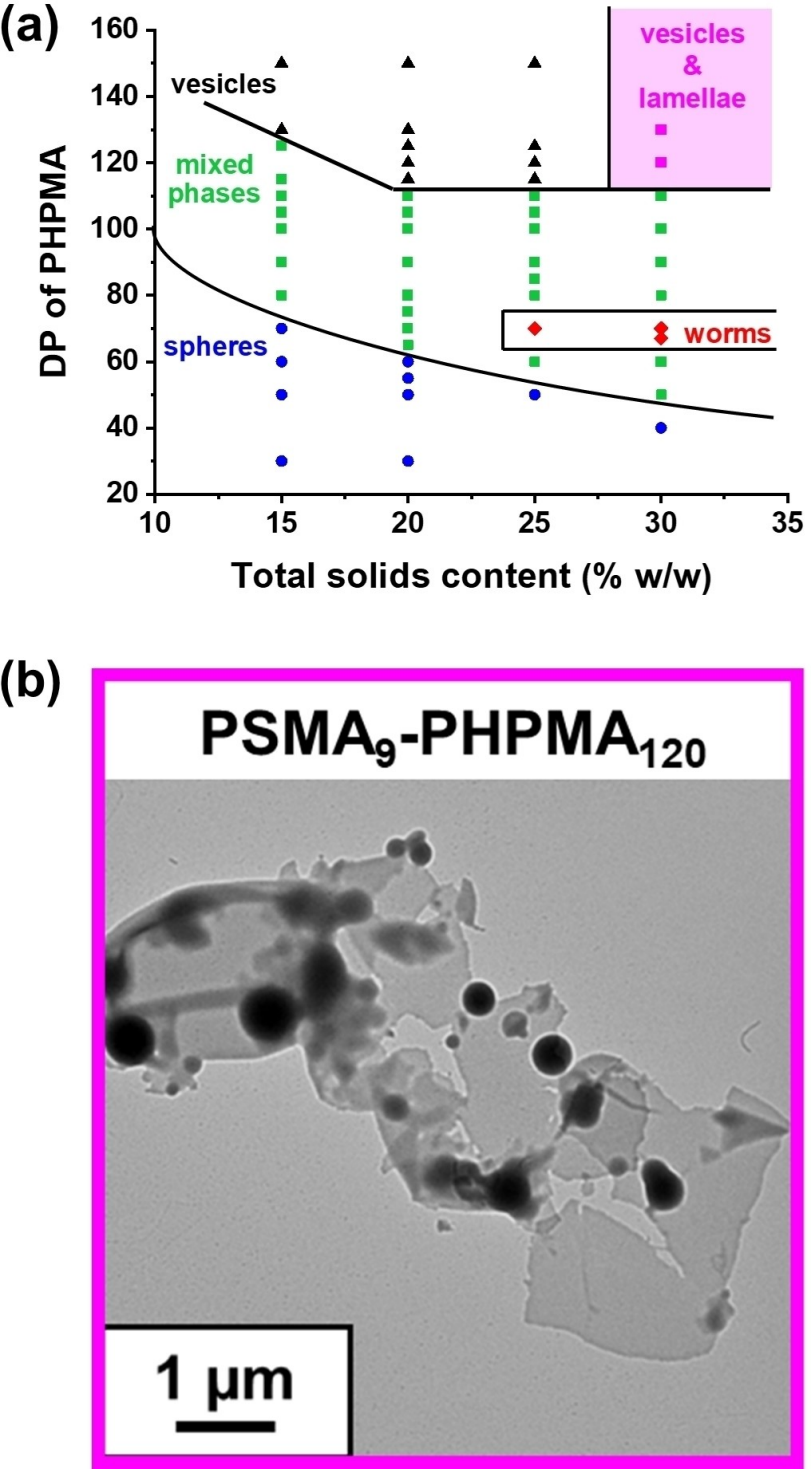
(a) Pseudo‐phase diagram constructed for PSMA_9_‐PHPMA_x_ diblock copolymer nano‐objects prepared by RAFT dispersion polymerization of HPMA in mineral oil using a PSMA_9_ precursor and T21 s initiator at 90 °C ([PSMA9]/[T21 s] molar ratio=5.0). [Black triangles (▴), red diamonds (⧫) and blue circles (•) represent pure vesicles, worms and spheres, respectively. Green squares (▪) correspond to either sphere/worm or worm/vesicle mixed phases, whereas purple squares (▪) represent a vesicle/lamellae mixed phase]. (b) Representative TEM image obtained for a mixed phase comprising PSMA_9_‐PHPMA_120_ lamellae and vesicles. Figure adapted from Ref. [66] with permission.

However, only a mixed phase comprising lamellae and vesicles could be accessed (see Figure 10) when targeting either PSMA_9_‐PHPMA_120_ or PSMA_9_‐PHPMA_130_ at 30 % w/w solids in mineral oil.

The acrylate analog of NMEP is *N*‐2‐(acryloyloxy)ethyl pyrrolidone (NAEP). This highly polar monomer has been used to prepare well‐defined PSMA_36_‐PNAEP_60–500_ spherical nanoparticles in *n*‐dodecane.^[67]^ This PISA formulation is a rare example of a *non‐aqueous* emulsion polymerization.^[49]^ A relatively fast rate of polymerization was observed for the synthesis of PSMA_36_‐PNAEP_60_ spherical nanoparticles at 20 % w/w solids in *n*‐dodecane at 90 °C (the NAEP monomer solubility is around 4.9 % v/v under such conditions). More than 99 % NAEP conversion was achieved within 1 h and a linear increase in *M*_n_ with conversion was confirmed by GPC analysis. The final diblock copolymer had an *M*_w_/*M*_n_ of 1.48; this indicates reasonably good

RAFT control despite the relatively high reaction temperature, which tends to promote chain transfer to polymer during the polymerization of acrylic monomers. A series of PSMA_36_‐PNAEP_60–500_ nanoparticles were analyzed by dynamic light scattering (DLS), small‐angle X‐ray scattering (SAXS) and transmission electron microscopy (TEM). DLS studies indicated a linear increase in z‐average diameter from 52 nm (PDI=0.10) to 261 nm (PDI=0.10) when increasing the PNAEP DP from 60 to 500. Differential scanning calorimetry (DSC) analysis of the dried diblock copolymers revealed PNAEP *T*_g_ values of −6 to −7 °C, indicating minimal molecular weight dependence. Such soft PNAEP cores led to partial deformation (flattening) of the PSMA_36_‐PNAEP_60–500_ nanoparticles during TEM grid preparation, leading to significant overestimation of the mean nanoparticle diameter compared to the SAXS and DLS data.

The PISA synthesis of epoxy‐functional poly(stearyl methacrylate)‐poly(glycidyl methacrylate) (PSMA‐PGlyMA) nanoparticles in mineral oil was reported by Docherty et al.^[31]^ A series of spherical nanoparticles were prepared via RAFT dispersion polymerization of GlyMA using either PSMA_13_ or PSMA_18_ as a steric stabilizer. The particle diameter could be varied between 21 and 86 nm by adjusting the target DP for the core‐forming PGlyMA block between 50 and 400. Chemical stability studies revealed that only 9 % of the epoxy groups were lost (presumably via reaction with trace water) over a 16‐week period when such nanoparticle dispersions were stored at ambient temperature. This was significantly less than that reported for PGlyMA‐core nanoparticles prepared in aqueous media (27 % over a 12‐week period).^[69]^ Post‐polymerization modification of PSMA_13_‐PGlyMA_375_ nanoparticles via amine‐epoxy chemistry was also demonstrated using *N*‐methylaniline as a model compound.^[31]^ In a follow‐up study, a relatively short PSMA_9_ stabilizer block was used to prepare the analogous epoxy‐functional worms and vesicles.^[41]^

**Figure 11:**
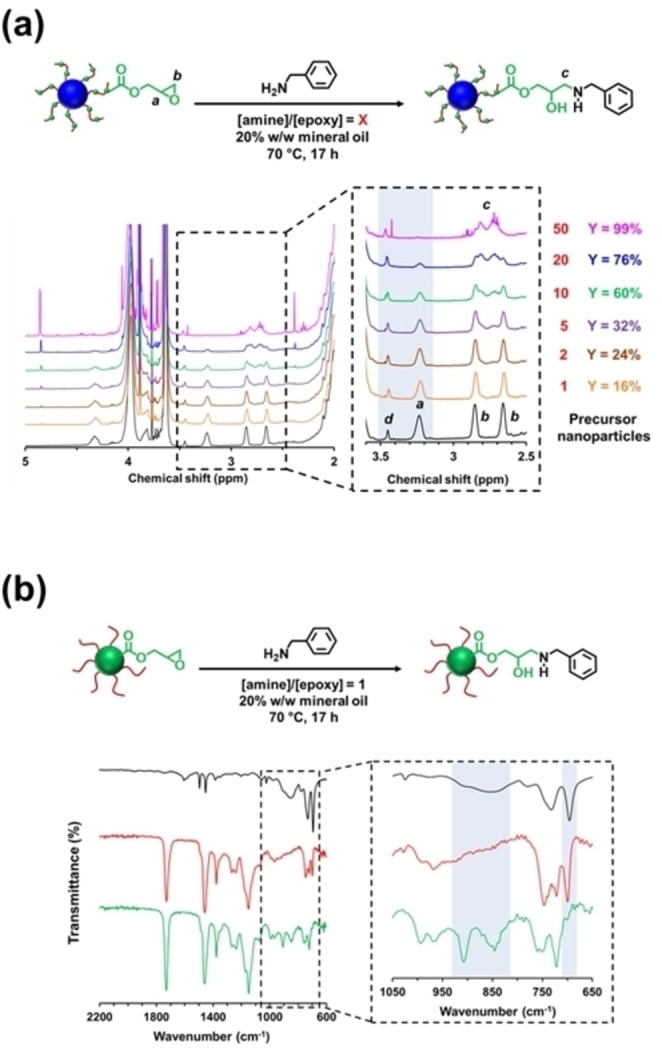
(a) ^1^H NMR spectra recorded in CD_2_Cl_2_ for P(LMA_50_‐*stat*‐GlyMA_9_)‐PMMA_67_ before (black spectrum) and after reaction with benzylamine at 70 °C for 17 h at 20 % w/w solids when using an [amine]/[epoxy] molar ratio (X) of 1 (orange spectrum), 2 (brown spectrum), 5 (purple spectrum), 10 (green spectrum), 20 (blue spectrum) and 50 (pink spectrum), respectively. Expansion of the 2.5–3.6 ppm region confirms the systematic loss of epoxy groups (note the gradual attenuation of methine signal *a* and the concomitant evolution of a new methylene signal *c* on increasing the [amine]/[epoxy] molar ratio. In each case, the epoxy loss (Y%) was determined by comparing the satellite signal *d* assigned to the PMMA backbone at 3.43–3.47 ppm to the methine signal *a* assigned to the epoxide ring at 3.12–3.30 ppm. (b) FT‐IR spectra recorded for PLMA_63_‐PGlyMA_89_ diblock copolymer prior to functionalization (green spectrum), after functionalization with benzylamine (red spectrum), and benzylamine alone (black spectrum). The reaction conditions used are summarized in the corresponding chemical reaction. Figure adapted from Ref. [68] with permission.

Recently, the post‐polymerization modification of two types of epoxy‐functional spherical nanoparticles was examined by György et al.^[68]^ More specifically, epoxy groups were either located within the nanoparticle cores (e.g., PLMA_63_‐PGlyMA_8_ nanoparticles prepared via RAFT dispersion polymerization of GlyMA) or within the steric stabilizer chains (e.g., P(LMA_50_‐*stat*‐GlyMA_9_)‐PMMA_67_) nanoparticles prepared using a statistical copolymer precursor comprising LMA and GlyMA), see Figure 11. Benzylamine was chosen as a model reagent to compare the reactivity of this pair of epoxy‐functional nanoparticles. For the PLMA_63_‐PGlyMA_89_ spheres, an [amine]/[epoxy] molar ratio of unity was sufficient to react all the epoxy groups, whereas the P(LMA_50_‐*stat*‐GlyMA_9_)‐PMMA_67_ spheres required a fifty‐fold excess of benzylamine for complete reaction. This striking difference was attributed to the relatively low molar concentration of the epoxy groups in the latter case (0.52 mol dm^−3^ vs. 0.07 mol dm^−3^). For the P(LMA_50_‐*stat*‐GlyMA_9_)‐PMMA_67_ spheres_,_ the degree of functionalization could be assessed by ^1^H NMR spectroscopy (Figure 11a). Unfortunately, this technique proved to be unsuitable for monitoring the extent of reaction for the PLMA_63_‐PGlyMA_89_ nanoparticles because epoxy ring‐opening is accompanied by cross‐linking side‐reactions. Instead, the extent of reaction for this system was evaluated using FT‐IR spectroscopy (Figure 11b). Ring‐opening of the epoxy groups by water to obtain hydroxyl‐functional nanoparticles was also examined. Heating a 20 % w/w dispersion of P(LMA_50_‐*stat*‐GlyMA_9_)‐PMMA_67_ nanoparticles in mineral oil at 110 °C in the presence of a trace amount of water was sufficient to ring‐open all of the epoxy groups, whereas derivatization of the PLMA_63_‐PGlyMA_89_ nanoparticles required the use of 50 % v/v aqueous acetic acid at the same temperature.^[68]^

### 2.4 Thermoresponsive Block Copolymer Nano‐objects

**Figure 12:**
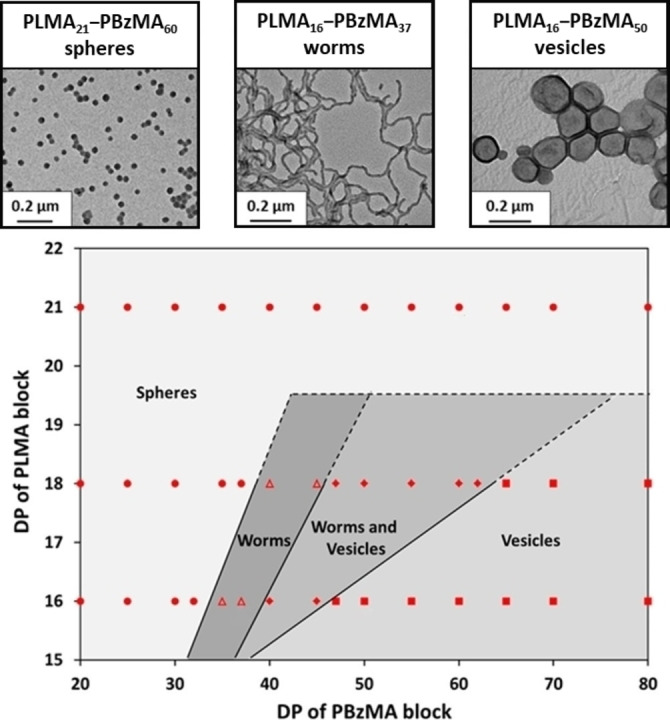
Phase diagram constructed for PLMA_x_‐PBzMA_y_ diblock copolymer nano‐objects prepared by RAFT dispersion polymerization of BzMA in *n*‐dodecane using an AIBN initiator at 70 °C when targeting 20 % w/w solids and systematically varying the PLMA and PBzMA DPs. Copolymer morphologies were assigned by TEM analysis. Figure is adapted from Ref. [63] with permission.

It is well‐known that poly(2‐hydroxypropyl methacrylate)‐based worms and vesicles prepared via aqueous PISA can undergo either worm‐to‐sphere, vesicle‐to‐worm or vesicle‐to‐sphere transitions on cooling below ambient temperature.[^70^, ^71^, ^72^] In 2013, Fielding et al. were the first to report the synthesis of well‐defined diblock copolymer worms and vesicles via RAFT dispersion polymerization of BzMA in *n*‐heptane using an oil‐soluble PLMA precursor.^[36]^ A year later, the same team reported the first PISA synthesis of *thermoresponsive* nano‐objects in non‐polar media.^[63]^ This was achieved by constructing a pseudo‐phase diagram for PISA syntheses conducted at 20 % w/w solids in *n*‐dodecane to aid the identification of a pure phase comprising highly anisotropic PLMA‐PBzMA worms (see Figure 12).^[63]^ This systematic approach established that a sufficiently short PLMA stabilizer block (PLMA DP≤21) was required to access higher order morphologies (e.g., worms or vesicles).

**Figure 13:**
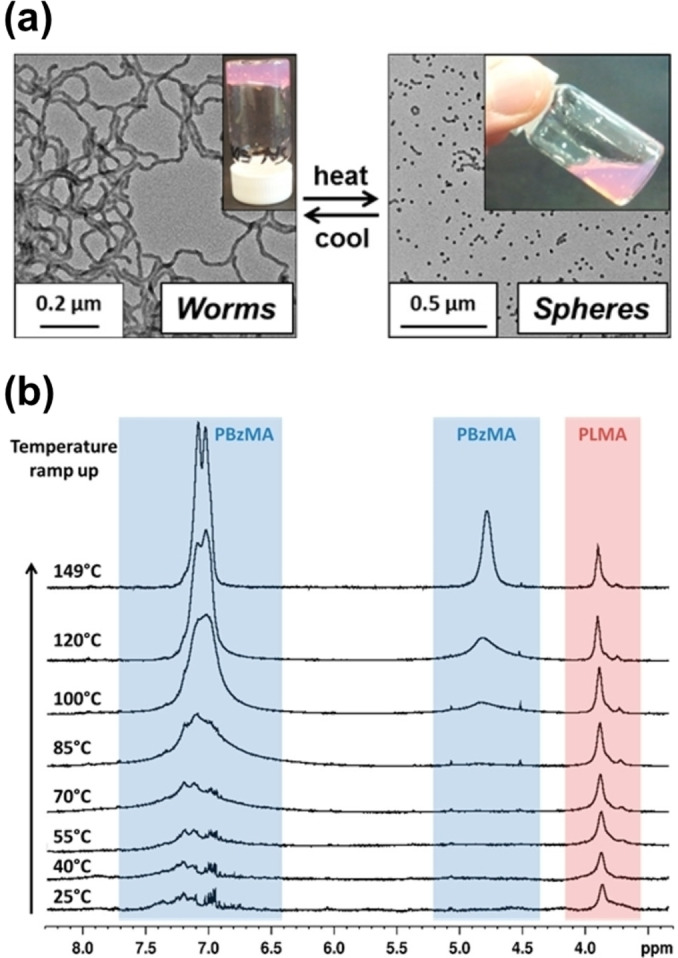
(a) Worm‐to‐sphere transition observed on heating a 20 % w/w dispersion of PLMA_16_‐PBzMA_37_ worms up to 90 °C in *n*‐dodecane. TEM analysis indicated reversible behaviour, with reconstituted worms being formed on cooling to 20 °C. (b) Variable‐temperature ^1^H NMR spectra recorded for 5.0 % w/w PLMA_16_‐PBzMA_37_ diblock copolymer worms in *d*_26_‐dodecane. Figure adapted from Ref. [63] with permission.

A 20 % w/w dispersion of PLMA_16_‐PBzMA_37_ worms in *n*‐dodecane formed a transparent free‐standing gel at 20 °C owing to the formation of a percolating 3D network comprising multiple inter‐worm contacts.^[73]^ However, degelation occurred on heating this worm gel up to 90 °C. TEM studies confirmed that this is the result of a worm‐to‐sphere transition (see Figure 13a) because isotropic spheres interact with each other much less efficiently than highly anisotropic worms.^[63]^ SAXS studies confirmed that this transition is more or less reversible at 20 % w/w solids but irreversible behaviour was observed at lower copolymer concentration (≤5 % w/w). This difference was explained in terms of the reduced probability of the many sphere‐sphere fusion events required to reconstitute the original worms—undoubtedly a highly cooperative process.^[63]^ Variable temperature SAXS studies indicated a gradual reduction in the mean worm contour length on heating, which reduces the multiple inter‐worm contacts that cause gelation. Variable temperature rheology studies indicated that degelation occurred at a relatively low temperature (∼47 °C). This is because it is not necessary to convert all the worms into spheres: if the mean worm length is reduced below that required for the percolation threshold this is sufficient to induce degelation.

Indeed, SAXS studies indicated that a purely spherical morphology was only obtained after heating the copolymer dispersion up to 160 °C. Moreover, SAXS and TEM studies also revealed that a mixture of short worms and spheres is formed at intermediate temperatures. Thus the mechanism for the worm‐to‐sphere transition was postulated to involve sequential budding of spheres from worm ends, rather than random worm scission.^[63]^ Variable temperature ^1^H NMR spectroscopy studies on a 5 % w/w dispersion of PLMA_16_‐PBzMA_37_ worms diluted in *d*_26_‐dodecane confirmed that partial solvation of the oil‐insoluble PBzMA block occurred on heating (see Figure 13b), which provided further physical insight regarding the nature of this thermally‐induced morphological transition. If *uniform* plasticization of the core‐forming PBzMA block occurred at elevated temperature this would increase its effective volume fraction and hence result in a *higher* packing parameter. This scenario incorrectly predicts a worm‐to‐vesicle transition. Instead, the worm‐to‐sphere transition is attributed to *surface* plasticization of the worms. In essence, this means that only those BzMA repeat units located nearest to the PLMA stabilizer block become solvated. Thus the effective volume fraction of the stabilizer block increases, which *lowers* the packing parameter and hence accounts for the observed morphological transition.^[63]^

Subsequently, a similar worm‐to‐sphere transition upon heating was reported by Lowe et al. for PSMA_18_‐PPPMA_71_ worms prepared at 30 % w/w solids in *n*‐octane^[52]^ or PSMA_19_‐PPPMA_85_ worms prepared at 20 % w/w solids in *n*‐tetradecane (where PPPMA denotes poly(phenylpropyl methacrylate).^[74]^ Moreover, Rymaruk et al. observed a thermoreversible worm‐to‐sphere transition for polydimethylsiloxane‐poly(2‐(dimethylamino)ethyl methacrylate) (PDMS_66_‐PDMA_100_) worms prepared in decamethylcyclopentasiloxane (D5) silicone oil.^[50]^

**Figure 14:**
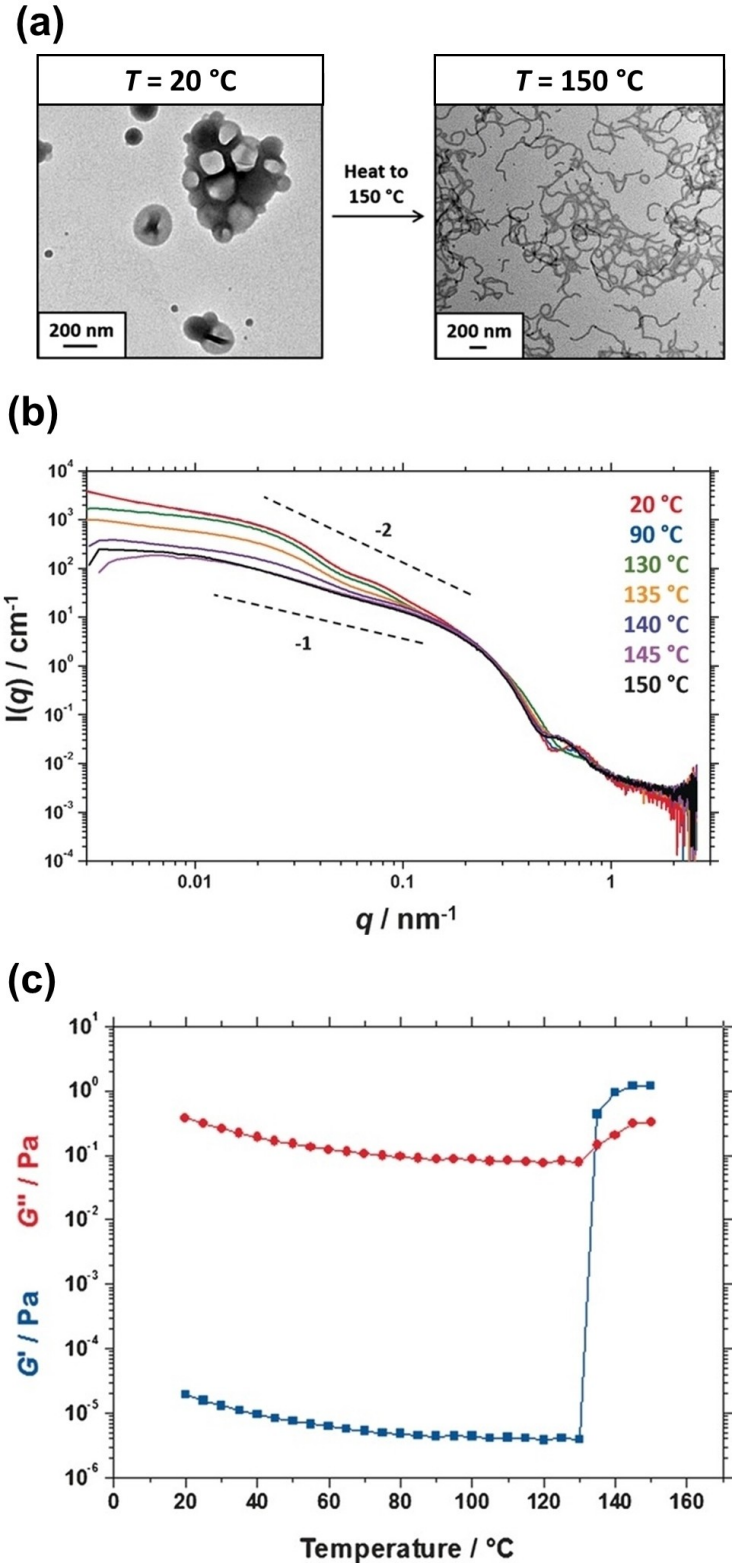
(a) Transmission electron micrographs obtained for 0.1 % w/w PSMA_13_‐PBzMA_96_ vesicles at 20 °C and the highly anisotropic worms formed by the same vesicle dispersion on heating up to 150 °C. (b) Variable‐temperature small‐angle X‐ray scattering (SAXS) patterns recorded for a 5.0 % w/w dispersion of PSMA_13_‐PBzMA_96_ nano‐objects in mineral oil. Gradients of −2 and −1 are shown as a guide to the eye. (c) Dependence of the storage modulus (*G*′, blue data) and loss modulus (*G*′′, red data) observed for a 10 % w/w dispersion of PSMA_13_‐PBzMA_96_ nanoparticles in mineral oil on heating from 20 to 150 °C. Data were obtained at 1.0 % strain using an angular frequency of 10 rad s^−1^, with a heating rate of 2 °C min^−1^. Figure adapted from Ref. [75] with permission.

The first example of a vesicle‐to‐worm transition was reported by Derry et al. for PSMA_13_‐PBzMA_96_ vesicles prepared directly in mineral oil (see Figure 14a).^[75]^ This morphology transition occurred on heating from 20 to 150 °C and variable temperature ^1^H NMR spectroscopy studies indicated partial solvation of the oil‐insoluble PBzMA block, which is consistent with a surface plasticization mechanism. In this case, variable temperature SAXS studies confirmed the gradual change in morphology (see Figure 14b). Moreover, mean aggregation numbers calculated for the initial vesicles and final worms suggested that, on average, each vesicle dissociated to produce three worms. Oscillatory rheology studies indicated an increase in the storage modulus (*G′*) by five orders of magnitude above 135 °C (see Figure 14c), which was determined to be the critical gelation temperature (CGT).

In a follow‐up study, Dorsman et al. reported that heating essentially the same PSMA‐PBzMA vesicles up to 180 °C produced a worm‐to‐sphere transition after the initial vesicle‐to‐worm transition.^[76]^ Moreover, statistical copolymerization of *n*‐butyl methacrylate (BuMA) with BzMA to form the core‐forming block enabled the critical temperature at which these morphology transitions occur to be tuned simply by adjusting the comonomer molar ratio. This is because PBuMA has a relatively low *T*_g_ compared to PBzMA (20 °C vs. 54 °C). For example, the vesicle‐to‐worm transition was observed at 167 °C for PSMA_14_‐PBzMA_125_ vesicles but occurred at 109 °C for PSMA_14_‐P(0.5BzMA‐*stat*‐0.5BuMA)_130_ vesicles. Further heating of the latter dispersion produced a mixture of short worms and spheres at 130 °C and a purely spherical morphology was obtained at 180 °C. The worm‐to‐sphere transition led to a sharp reduction in both the storage modulus and the dispersion viscosity.

In this context, it is also worth mentioning that a partial *worm‐to‐vesicle* transition has been reported for both PSMA_9_‐PGlyMA_75_ and PSMA_9_‐PHPMA_70_ worms prepared in mineral oil.[^41^, ^66^] Such unusual thermal behaviour implies an increase in the packing parameter, i.e., the effective volume fraction of the core‐forming block must increase relative to that of the stabilizer block. In principle, this could occur via uniform solvation of the core‐forming block but this seems rather unlikely given that mineral oil is likely to remain a poor solvent for the PGlyMA (or PHPMA) block at elevated temperature. Alternatively, the PSMA_9_ block may become less solvated at elevated temperature and hence occupy a smaller volume relative to that at 20 °C. Further mechanistic studies would be required to provide a satisfactory physical explanation for such unexpected morphological transitions.[^41^, ^66^]

In 2018, Derry et al. reported the synthesis of poly(behenyl methacrylate)‐poly(benzyl methacrylate) (PBeMA‐PBzMA) nanoparticles in mineral oil.^[77]^ Targeting PBeMA_37_‐PBzMA_x_ at 20 % w/w solids resulted in the formation of relatively transparent colloidal dispersions at the synthesis temperature of 90 °C but turbid pastes were obtained on cooling to ambient temperature. Combined SAXS, WAXS and DSC studies indicated crystallization of the pendent behenyl (C_22_H_45_) groups on the steric stabilizer chains, which occurred both within the individual nanoparticles and between neighbouring nanoparticles. Reheating the turbid paste up to 50 °C led to complete redispersion of the flocculated nanoparticles as judged by turbidimetry and SAXS analysis.

Similar colloidal instability on cooling was reported by Gibson et al. for poly(*tert*‐octyl acrylamide)‐poly(*N,N*‐dimethylacrylamide) (POAA_85_‐DMAC_x_) spherical nanoparticles prepared in certain *n*‐alkanes.^[39]^ This system was the first all‐acrylamide PISA formulation reported for non‐polar media and both high DMAC conversions and reasonably good RAFT control (*M*_w_/*M*_n_≤1.42) were achieved. Colloidally stable nanoparticles were obtained at 20 °C in *n*‐heptane, *n*‐octane or *n*‐decane, whereas employing *n*‐dodecane, *n*‐tetradecane or *n*‐hexadecane led to nanoparticle flocculation on cooling from the synthesis temperature (70 °C) to 20 °C. In this case, this is simply because the POAA_85_ stabilizer chains exhibit upper critical solution temperature (UCST)‐type behaviour in higher *n*‐alkanes, which leads to the loss of steric stabilization at 20 °C. Thus, unlike the PBeMA‐PBzMA nanoparticles, this system does not involve partial crystallization between steric stabilizer chains.

Recently, Gardoni et al. reported the synthesis of nanoparticles comprising poly(di(ethylene glycol) methyl ether methacrylate) (PDEGMA) cores using a PLMA precursor in a 1 : 1 mixture of *n*‐decane and toluene.^[78]^ Interestingly, PDEGMA exhibited UCST‐type behaviour in this binary solvent. More specifically, colloidally stable nanoparticles (spheres or worms) were obtained below the cloud point (*T*_cp_) of this core‐forming block. Moreover, *T*_cp_ increased linearly when adjusting the PDEGMA DP from 400 to 1200. Thus the critical temperature at which nanoparticle formation occurs can be readily tuned.

### 2.5 Non‐methacrylic Steric Stabilisers

Non‐methacrylic polymers such as polyacrylates,[^33^, ^35^, ^38^, ^79^] polyacrylamides,^[39]^ polydimethylsiloxane[^48^, ^49^, ^50^] or polyolefins[^80^, ^81^] can also provide effective steric stabilization for nano‐objects in non‐polar media. The earliest examples of PISA syntheses conducted in non‐polar media were reported by Charleux et al. in 2007^[35]^ and 2010.[^33^, ^79^] In this case, an all‐acrylic formulation was employed in which poly(2‐ethylhexyl acrylate) served as the steric stabilizer block in *iso*‐dodecane and poly(methyl acrylate) was used as the core‐forming block to produce solely spherical nanoparticles.[^33^, ^35^, ^79^] Subsequently, Ratcliffe et al. reported an all‐acrylic PISA formulation that provided access to higher order morphologies.^[38]^ More specifically, the synthesis of poly(lauryl acrylate)‐poly(benzyl acrylate) (PLA‐PBzA) nano‐objects was studied in three different solvents: *n*‐heptane, *n*‐dodecane, or *iso*‐hexadecane. Because of the low *T*_g_ of the core‐forming PBzA block, cryo‐TEM was required to assign the copolymer morphologies. A detailed pseudo‐phase diagram was constructed when using a PLA_14_ precursor to target PBzA DPs of 50–100 at 5–25 % w/w solids in *n*‐heptane. Furthermore, a critical gelation concentration (CGC) as low as 2.5 % w/w was determined for PLA_14_‐PBzA_60_ worms, which could be prepared at up to 40 % solids in *n*‐dodecane using a convenient one‐pot protocol.^[38]^

Lopez‐Oliva et al. reported the first use of polydimethylsiloxane as a steric stabilizer for PISA syntheses. In this case, a commercially available monohydroxy‐terminated precursor (DP=66) was modified via Steglich esterification using a carboxylic acid‐functionalized RAFT agent (PETTC, see Figure 2).^[48]^ The resulting trithiocarbonate‐functionalized PDMS_66_ chains were employed for the RAFT dispersion polymerization of BzMA at 70 °C in *n*‐heptane. This PISA formulation produced a wide range of well‐defined spheres and vesicles. However, only a *single* diblock copolymer composition (PDMS_66_‐PBzMA_80_) resulted in the formation of pure worms, which could only be obtained when targeting either 25 % or 30 % w/w solids.

An interesting PISA formulation based on a polyolefin‐based RAFT agent was reported by Darmau et al.^[80]^ More specifically, a monohydroxyl‐functionalized hydrogenated polybutadiene (PhBD) precursor was subjected to Steglich esterification using PETTC to produce the corresponding PhBD RAFT agent. Subsequently, a series of PhBD_80_‐PBzMA_30–300_ nano‐objects were produced when targeting 25–45 % w/w solids in *n*‐dodecane at 90 °C. GPC analysis of aliquots extracted during the synthesis of PhBD_80_‐PBzMA_200_ at 25 % w/w solids indicated that a significant fraction of the PhBD precursor remained unreacted even at 60 % BzMA conversion. However, this macromolecular RAFT agent was gradually consumed as the polymerization proceeded further, resulting in diblock copolymers with relatively narrow molecular weight distributions (*M*_w_/*M*_n_≤1.22). Kinetically‐trapped spheres were invariably obtained at or below 25 % w/w solids: pure worms and vesicles were only obtained from PISA syntheses conducted at 40–45 % w/w solids.

**Figure 15:**
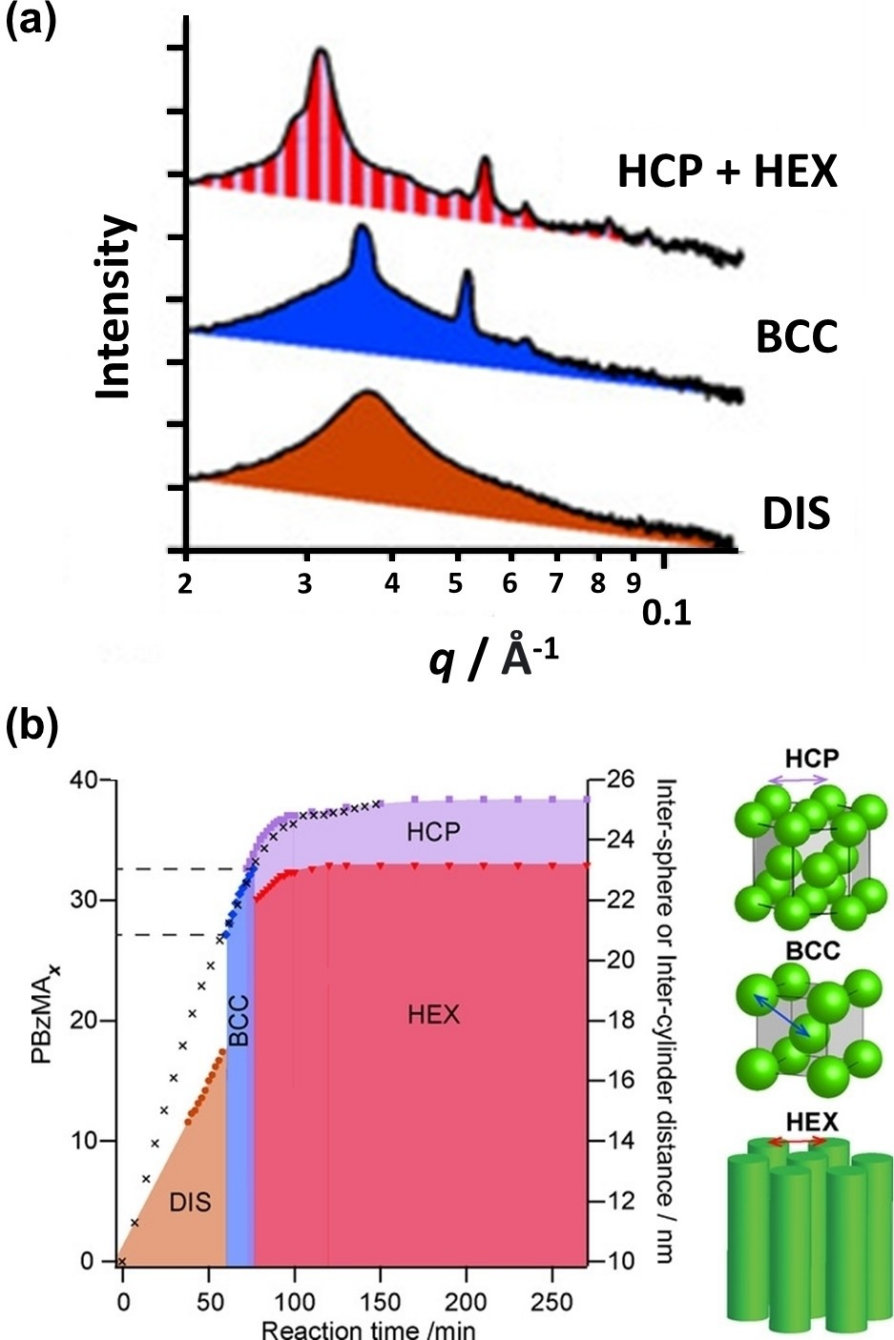
(a) Selected 1D SAXS patterns recorded during the synthesis of PhBD_80_‐PBzMA_40_ copolymer at 40 % w/w/solids in *n*‐dodecane at 90 °C. DIS indicates a disordered array of micelles which most likely possess a pseudo‐spherical morphology. BCC, HCP and HEX denote body‐centered cubic, hexagonally close‐packed phases and hexagonally packed cylinders, respectively. (b) Reaction phase map recorded during the PISA synthesis of PhBD_80_‐PBzMA_40_ diblock copolymer nano‐objects at 40 %w/w solids in *n*‐dodecane. Colored symbols denote domain spacings within different phases calculated from time‐resolved SAXS data, while black crosses indicate the mean degree of polymerization (*x*) of the insoluble PBzMA block calculated from in situ ^1^H NMR studies. The two dashed lines shown on the left indicate the approximate time points at which the disorder‐order and order‐order phase transitions occur. The schematic representations illustrate the inter‐sphere distances for the HCP and BCC phases and the inter‐cylinder distance for HEX. The green spheres and cylinders represent the PBzMA cores of nano‐objects that form structured arrangements within a continuous phase comprising PhBD_80_ chains and *n*‐dodecane. Figure adapted from Ref. [81] with permission.

The same PhBD_80_‐PBzMA_y_ formulation was used to identify the formation of lyotropic phases during PISA (see Figure 15a).^[81]^

Time‐resolved SAXS studies were performed when targeting PhBD_80_‐PBzMA_40_ worms at 40 % w/w solids in *n*‐dodecane. Such experiments revealed a morphology evolution from molecularly dissolved copolymer chains to spheres to close‐packed spheres (either body‐centered cubic (BCC) or hexagonally close‐packed (HCP) phases) to a final mixture of HEX and HCP phases (where HEX denotes hexagonally‐packed cylinders ‐ or partially aligned worms ‐ and is the major phase). In situ ^1^H NMR experiments performed for the same PISA formulation provided the monomer conversion vs. time curve, from which the corresponding instantaneous diblock copolymer composition could be determined for each morphology (see Figure 15b). As far as we are aware, this is the only example of the self‐assembly of nano‐objects during PISA (in addition to the self‐assembly of the diblock copolymer chains). In principle, similar behaviour should be expected for many other PISA formulations, including aqueous formulations. However, it remains to be seen whether this is actually the case in practice.

### 2.6 PISA Syntheses in Silicone Oil

**Figure 16:**
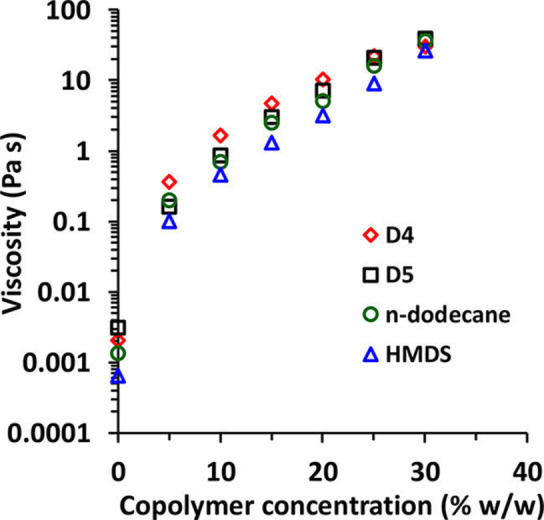
Concentration dependence of the solution viscosity (determined at a fixed shear rate of 10 s^−1^) for PDMS_66_‐PDMA_x_ diblock copolymer worms prepared in either D5 silicone oil (open black squares), D4 silicone oil (open red diamonds), HMDS (open blue triangles), or *n*‐dodecane (open green circles), where×varies between 91 and 110 depending on the solvent type. In each case, worms were prepared at an initial copolymer concentration of 30 % w/w solids and then sequentially diluted using the same solvent for viscosity measurements. Figure adapted from Ref. [49] with permission.

Silicone oils are non‐toxic, chemically inert, and non‐flammable: they offer many applications ranging from antifoaming agents^[82]^ to cosmetic formations.[^49^, ^83^] Rymaruk et al. reported the first PISA syntheses to be conducted in such solvents.[^49^, ^50^, ^54^] For example, a trithiocarbonate‐capped PDMS_66_ precursor^[48]^ was chain‐extended using 2‐(dimethylamino)ethyl methacrylate (DMA) in low‐viscosity silicone oils such as decamethylcyclopentasiloxane (D5), octamethylcyclotetra‐siloxane (D4) or hexamethyldisiloxane (HMDS).^[49]^ In addition to well‐defined spheres, these formulations provide convenient access to worms and vesicles, which was attributed to the relatively low *T*_g_ of ∼18 °C for the structure‐directing PDMA block. In striking contrast, only kinetically‐trapped spheres could be obtained when using BzMA, 2,2,2‐trifluoroethyl methacrylate (TFEMA), methyl methacrylate (MMA) or HPMA to generate the oil‐insoluble block. Rotational rheology experiments indicated that a 5 % w/w dispersion of PDMS_66_‐PDMA_100_ worms produced a sixty‐fold increase in solution viscosity relative to that for the corresponding pure solvent (see Figure 16).

Subsequently, the same worms were covalently stabilized using 1,2‐bis(2‐iodoethoxy)ethane.^[50]^ This bifunctional reagent quaternizes the tertiary amine groups on the DMA residues within the worm cores, thus introducing cross‐links via the Menshutkin reaction. Oscillatory rheology studies of 25 % w/w gels formed by the linear and core‐crosslinked PDMS_66_‐PDMA_100_ worms revealed that using a BIEE/DMA molar ratio of 0.15 increased the worm gel strength (*G′*) dramatically from 94 to 7855 Pa. Such derivatization also reduced the CGC from 12 % w/w for the linear worms to just 2 % w/w for the core‐crosslinked worms. This was attributed to the much greater stiffness (i.e., longer effective Kuhn length) of the latter nano‐objects. Unlike the linear PDMS_66_‐PDMA_100_ worms, the core‐crosslinked worms did not exhibit any thermoresponsive behaviour at elevated temperature.

In related work, poly(3‐[tris(trimethylsiloxy)silyl]propyl methacrylate)‐poly(benzyl methacrylate) (PSiMA‐PBzMA) diblock copolymer nano‐objects have been prepared via PISA syntheses conducted in D5.^[54]^

According to the pseudo‐phase diagram constructed for PSiMA_12_‐PBzMA_x_ nanoparticles (x≤200), varying the solids content between 5 % w/w and 20 % w/w provided access to a pure worm phase even at the lowest copolymer concentration (5 % w/w). This is rather unusual: this elusive morphology usually requires somewhat higher copolymer concentrations.[^36^, ^37^, ^52^, ^53^, ^74^, ^80^] Similarly, well‐defined vesicles could be obtained from syntheses performed at just 10 % w/w solids. Furthermore, GPC analysis of PSiMA_12_‐PBzMA_35_ spheres and PSiMA_12_‐PBzMA_55_ worms after six weeks storage at 20 °C indicated significant broadening of their molecular weight distributions. This long‐term chemical instability was tentatively attributed to hydroxyl impurities within the SiMA monomer.

### 2.7 Rheology Studies of Worm Gels

**Figure 17:**
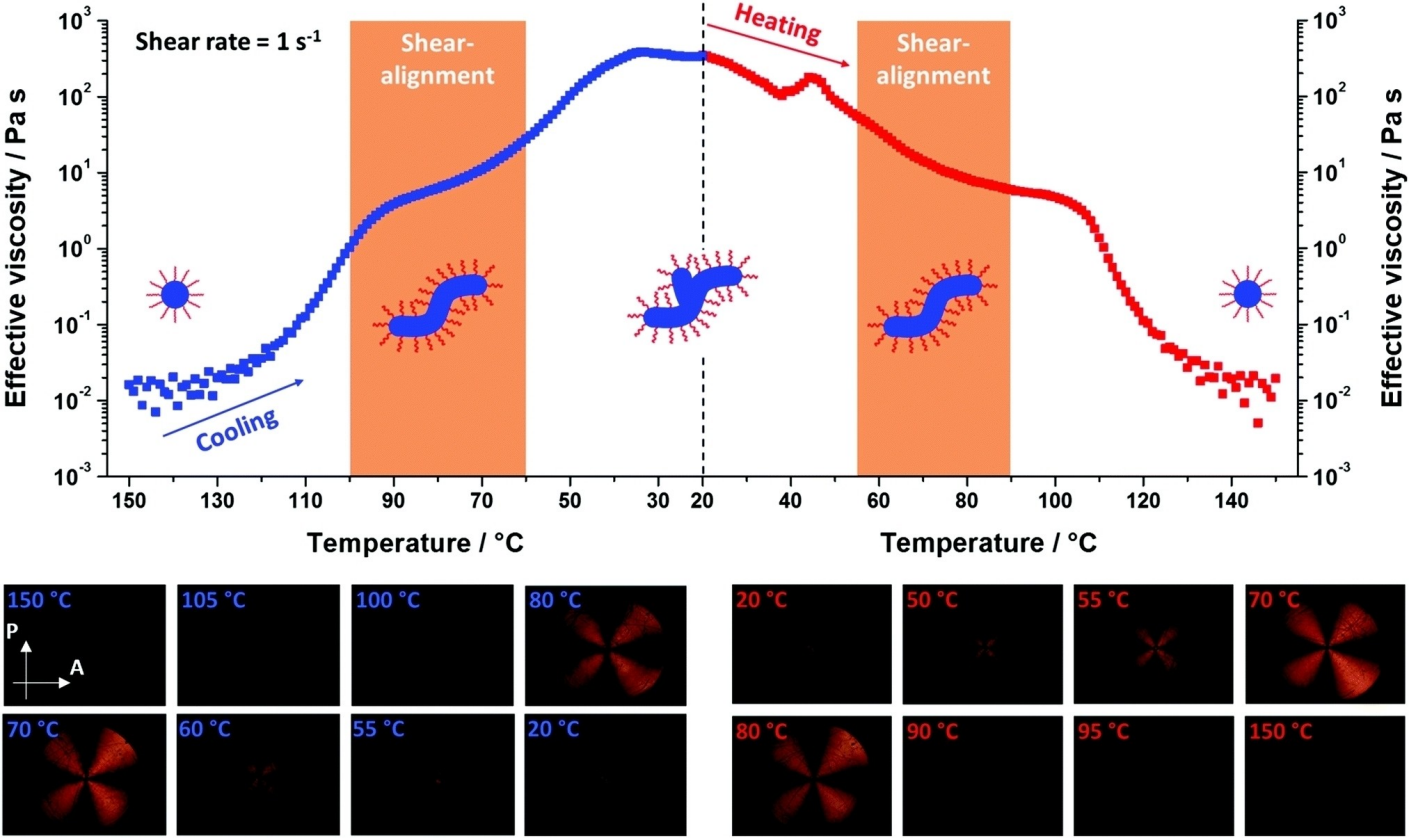
Dispersion viscosity‐temperature profile and corresponding polarized light images (PLIs) obtained for a 20 % w/w dispersion of PSMA_13_‐PBzMA_64_ nano‐objects on cooling from 150 °C to 20 °C (blue data, left of the dashed vertical line) and on heating from 20 °C to 150 °C (red data, right of the dashed vertical line) at a rate of 2 °C min^−1^ when employing a constant maximum (sample edge) shear rate of 1 s^−1^. Selected PLIs represent the sample birefringence observed at various temperatures. Arrows show the planes of polarization for the polarizer (P) and the analyzer (A), crossed at 90°. A Maltese cross motif indicates shear‐induced alignment of anisotropic objects, whereas its absence indicates either no alignment or no anisotropic objects. The diameter of the sample is 25 mm. Figure adapted from Ref. [84] with permission.

One important aspect of highly anisotropic diblock copolymer worms is their distinctive rheological behaviour. Derry et al. studied PSMA_13_‐PBzMA_64_ worms in mineral oil using a shear‐induced polarized light imaging (SIPLI) technique.^[84]^ Above a certain critical shear rate, highly anisotropic particles tend to align in the direction of flow, which results in shear‐thinning (i.e., significantly lower viscosity). Worm alignment was also studied as a function of temperature (see Figure 17). Like the PLMA_16_‐PBzMA_37_ worms described above, PSMA_13_‐PBzMA_64_ worms (prepared in mineral oil) exhibit a worm‐to‐sphere transition on heating from 20 to 150 °C. Thus the initial shear‐thinning anisotropic worms are converted into isotropic spherical nanoparticles, which behave as Newtonian fluids. A 20 % w/w dispersion of PSMA_13_‐PBzMA_64_ nanoparticles was monitored by SIPLI during a thermal cycle from 20 °C to 150 to 20 °C to determine the relationship between dispersion viscosity and shear alignment (see Figure 17). Between 20 and 60 °C, the dispersion had a relatively high viscosity owing to the presence of worms. Aligned linear worms were obtained at 80–110 °C, while further heating up to 150 °C produced a low‐viscosity dispersion of spherical nanoparticles.

**Figure 18:**
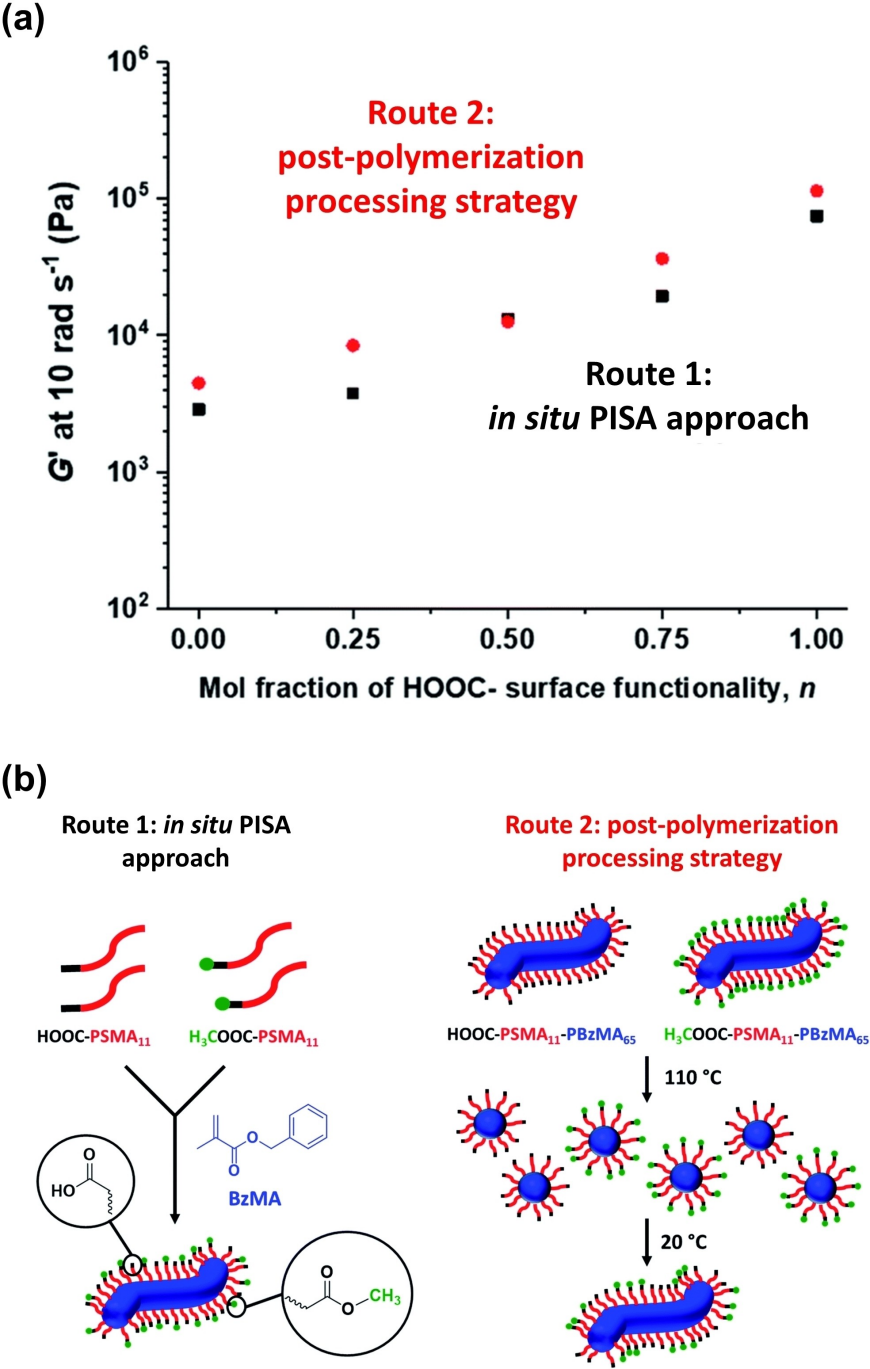
(a) Effect of varying the mole fraction of carboxylic acid end groups on the storage modulus, *G*′, for 20 % w/w PSMA_11_‐PBzMA_65_ worm gels prepared by Routes 1 and 2 [*G*′ data recorded at an angular frequency of 10 rad s^−1^]. (b) Schematic representation of the two synthetic routes used to prepare two series of PSMA‐PBzMA worms containing various proportions of carboxylic acid end groups. Both routes are based on the principle of entropic mixing. Route 1 utilizes a binary mixture of HOOC‐PSMA_11_ and H_3_COOC‐PSMA_11_ precursors during the RAFT dispersion polymerization of BzMA; this approach results in a statistical distribution of carboxylic acid end groups located at the outer surface of each sterically stabilized worm. Route 2 involves heating two ‘masterbatch’ 20 % w/w dispersions comprising HOOC‐PSMA_11_‐PBzMA_65_ and H_3_COOC‐PSMA_11_‐PBzMA_65_ worm gels up to 110 °C to induce a worm‐to‐sphere transition (and concomitant degelation) in each case. These two free‐flowing fluids of spherical nanoparticles were then mixed together in various proportions at 110 °C to produce the desired range of carboxylic acid/methyl ester molar ratios. On cooling to 20 °C, a sphere‐to‐worm transition occurs by 1D stochastic fusion of multiple (mixed) spheres to produce ‘hybrid’ segmented worms comprising spatially localized patches of steric stabilizer chains bearing carboxylic acid end groups. Figure adapted from Ref. [85] with permission.

The same PSMA‐PBzMA formulation was also used to design hydrogen‐bonded worm gels in *n*‐dodecane.^[85]^ This was achieved by using PETTC to prepare a carboxylic acid‐functionalized PSMA homopolymer (HOOC‐PSMA_11_) via RAFT solution polymerization. Approximately half of this precursor was then subjected to Steglich esterification using excess methanol to produce the corresponding methyl ester‐functionalized PSMA homopolymer (H_3_COOC‐PSMA_11_). When targeting the same PSMA_11_‐PBzMA_65_ worms, the HOOC‐PSMA_11_ precursor produced a much stronger physical gel (*G′* ∼114 kPa) than that obtained when using the H_3_COOC‐PSMA_11_ precursor (*G′* ∼4.5 kPa), see Figure 18a. This substantial (∼25‐fold) increase in *G′* was attributed to the formation of carboxylic acid dimers between neighbouring worms within the 3D percolating network. Introducing such carboxylic acid functionality into a diblock copolymer formulation is trivial because it simply requires a carboxylic acid‐based RAFT agent such as PETTC or DDMAT (see Figure 2). The authors showed that a series of worm gels with tunable gel strength can be readily prepared by using binary mixtures of carboxylic acid‐ and methyl ester‐capped PSMA_11_ precursors during PISA. Alternatively, a post‐polymerization processing strategy can be utilized to exploit the reversible worm‐to‐sphere transition exhibited by such worms. Thus, a binary mixture of acid‐ and ester‐functionalized spheres can be readily prepared by heating the two corresponding worm gels up to 110 °C to induce a worm‐to‐sphere transition in each case, followed by addition of one hot free‐flowing fluid to the other. Cooling to 20 °C leads to the formation of ‘hybrid’ segmented worms comprising spatially‐localized patches of carboxylic acid‐capped steric stabilizer chains (see Figure 18b).^[85]^ For either method, a similar progressive increase in gel strength (*G′*) was observed when increasing the mole fraction of carboxylic acid end‐groups see Figure 18a.

Very recently, Calabrese et al. used two different microfluidic set‐ups to compare the shear and extensional flow behaviour of relatively long, flexible PSMA_10_‐PBzMA_49_ worms with that of relatively short, stiff PSMA_10_‐PMMA_83_ worms in mineral oil.^[86]^ According to flow‐induced birefringence (FIB) analysis, the PSMA_10_‐PMMA_83_ worms behave as rigid rods under flow, whereas the more flexible PSMA_10_‐PBzMA_49_ worms undergo stretching and alignment much more efficiently under extensional flow than under shear flow. In principle, the latter worms may offer potential applications as additives for industrial formulations that experience extensional‐dominated flow (e.g., jetting, spraying or printing processes).

### 2.8 In Situ Studies During PISA Syntheses

**Figure 19:**
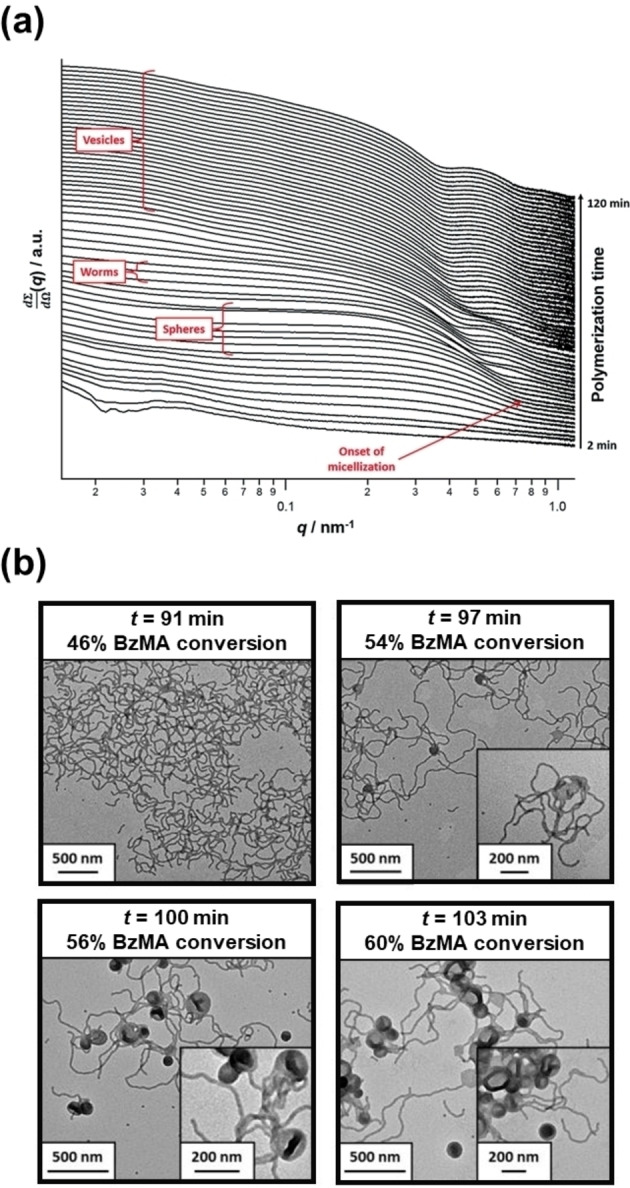
(a) In situ SAXS patterns recorded during the PISA synthesis of PSMA_13_‐PBzMA_150_ diblock copolymer vesicles prepared at 90 °C in mineral oil at 10 % w/w solids using a capillary cell. The onset of micellar nucleation is indicated by the red arrow. (b) TEM images recorded for 0.1 % w/w dispersions of PSMA_13_‐PBzMA_x_ nanoparticles obtained at various time points during the PISA synthesis of PSMA_13_‐PBzMA_150_ vesicles during the equivalent laboratory‐scale synthesis at 10 % w/w solids in mineral oil. A pure worm morphology is observed after 91 min, worms and octopi structures are observed after 97 min and worms, vesicles, octopi and jellyfish structures are observed after 100 min and 103 min, respectively. Figure adapted from Ref. [42] with permission.

Spectroscopic or scattering techniques have provided detailed information regarding polymerization kinetics, solvent plasticization of the insoluble block, morphology evolution or morphology transitions for either aqueous and/or alcoholic PISA formulations.[^87^, ^88^, ^89^, ^90^, ^91^, ^92^] Such techniques have also been used to study various PISA formulations in non‐polar media. For example, Derry et al.^[42]^ conducted in situ SAXS studies during the synthesis of PSMA_13_‐PBzMA_150_ vesicles at 10 % w/w solids in mineral oil within a capillary cell. As expected, a gradual evolution in copolymer morphology from molecularly‐dissolved chains to spheres to worms to vesicles was observed (see Figure 19a). Comparing the kinetic data estimated for this PISA formulation with the corresponding pseudo‐phase diagram constructed for PSMA_13_‐PBzMA_x_ nano‐objects indicated that the critical PBzMA DP boundaries for pure copolymer morphologies (spheres, worms or vesicles) were in remarkably good agreement. Moreover, *postmortem* DLS, TEM and SAXS analyses indicated that, once vesicles were formed at around 72 % BzMA conversion, their mean membrane thickness increased monotonically with monomer conversion during the latter stages of the polymerization.

Furthermore, the *overall* vesicle diameter remained constant. Thus, the vesicle lumen volume *decreases* during the polymerization, which implies an ‘inward growth’ mechanism for such vesicles. Similar observations were reported by Warren et al. for PGMA‐PHPMA vesicles prepared via aqueous PISA so at first sight this appears to be a *generic* mechanism for the growth of diblock copolymer vesicles during PISA.^[93]^ However, Tan et al. recently reported a different growth mechanism for PGMA_62_‐PHPMA_600–1400_ vesicles, which were prepared via photoinitiated RAFT aqueous dispersion polymerization of HPMA using a sodium phenyl‐2,4,6‐trimethylbenzoylphosphinate (SPTP) photocatalyst and visible light irradiation (λ=405 nm) at 60 °C.^[94]^

The same PSMA_13_‐PBzMA_150_ vesicles were also prepared at 10 % w/w solids in mineral oil on a sufficiently large scale to enable the periodic extraction of aliquots from the reaction mixture for TEM analysis. For the first time, intermediate morphologies such as octopi and jellyfish (see Figure 19b) could be identified during the evolution from worms to vesicles, similar to observations made by Blanazs and co‐workers for an aqueous PISA formulation targeting PGMA_47_‐PHPMA_200_ vesicles.^[95]^

**Figure 20:**
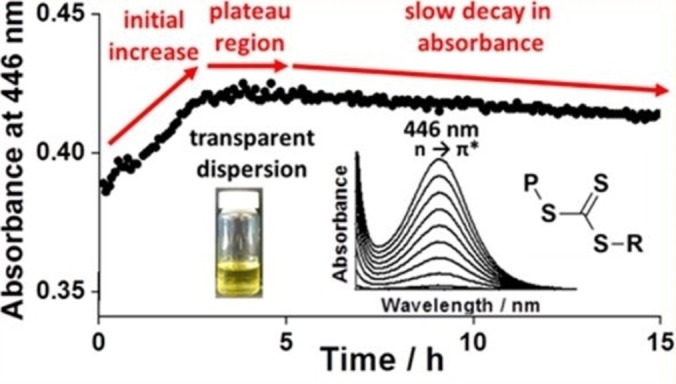
Typical absorbance versus time plot obtained from in situ visible absorption spectroscopy studies of the RAFT dispersion polymerization of TFEMA in *n*‐tetradecane using a PSMA_12_ stabilizer block at 70 °C, where the weak absorption band at 446 nm is assigned to the trithiocarbonate RAFT end groups. Matching the refractive index of the PTFEMA nanoparticle cores to that of the solvent enables high‐quality visible absorption spectra to be recorded (see inset) even when targeting 30 % w/w solids. The constant absorbance at 446 nm observed between 3 h and 5 h indicates excellent RAFT chain‐end stability under monomer‐starved conditions, with only a relatively slow decay in absorbance being observed thereafter. Figure adapted from Ref. [43] with permission.

Cornel et al. reported the rational synthesis of highly transparent colloidal dispersions comprising spherical nanoparticles via RAFT dispersion polymerization of TFEMA using a PSMA_12_ precursor.^[43]^ More specifically, PSMA_12_‐PTFEMA_98_ nanoparticles of 33 nm diameter were prepared at 30 % w/w solids in *n*‐tetradecane at 70 °C. This solvent has the same refractive index (approximately 1.42) as the PTFEMA core‐forming block at the synthesis temperature, which ensured that more than 99 % transmittance was achieved throughout the entire polymerization. Such transparency enabled this PISA formulation to be monitored by in situ visible absorption spectroscopy by focusing on the relatively weak n→π* transition band assigned to the trithiocarbonate (PETTC) RAFT agent at 446 nm. Initially, a gradual increase in absorbance was observed owing to volume contraction of the reaction solution as the low‐density TFEMA monomer (*ρ*=1.18 g cm^−3^) was converted into high‐density PTFEMA (*ρ*=1.47 g cm^−3^). This dilatometric effect was exploited to determine the kinetics of the TFEMA polymerization, which was validated by performing in situ ^19^F NMR spectroscopy studies. Once this polymerization had ceased, the absorbance remained constant for 2 h at 70 °C, which indicated remarkably high RAFT chain‐end fidelity under monomer‐starved conditions (see Figure 20). Interestingly, when using a dithiobenzoate‐based chain transfer agent (CPDB) to produce highly transparent PSMA_12_‐PTFEMA_98_ spheres, premature loss of the RAFT chain‐ends occurred during the RAFT dispersion polymerization of TFEMA at 90 °C, which prevented similar kinetic studies.^[96]^ Although RAFT polymerization offers many synthetic advantages, the resulting copolymers are often unsuitable for various potential applications (e.g., cosmetics or personal care products) owing to the color and malodor conferred by the sulfur‐based chain‐ends. Cornel et al. showed that the extent of chain‐end removal could be monitored in real time via in situ visible absorption spectroscopy by adding excess initiator to the nanoparticle dispersion after the TFEMA polymerization was complete. More than 98 % of the RAFT chain ends were removed within 8 h at 70 °C when using an [initiator]/[trithiocarbonate] molar ratio of either 7.5 or 10.^[43]^

**Figure 21:**
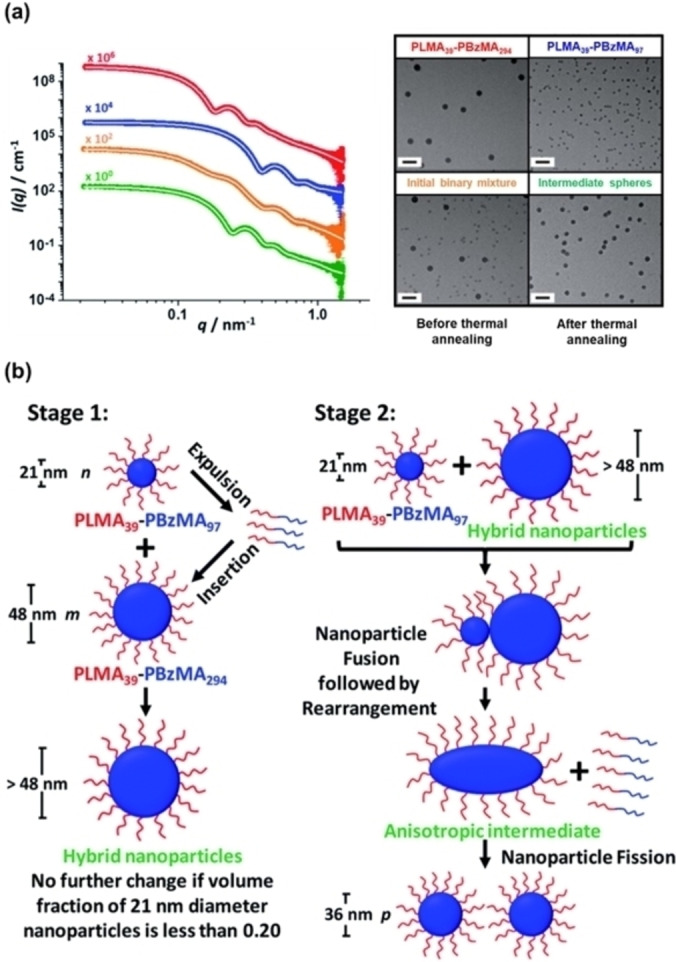
(a) SAXS patterns (and corresponding TEM images) recorded at 25 °C for 1.0 % w/w dispersions of PLMA_39_‐PBzMA_294_ spheres (red data) and PLMA_39_‐PBzMA_97_ spheres (blue data), a 1.0 % w/w equivolume binary mixture of these two initial dispersions prior to thermal annealing (orange data), and the final hybrid nanoparticles formed after thermal annealing of the same binary mixture at 150 °C for 1 h (green data). White traces indicate the best fits to the data obtained when using a spherical micelle model.^[97]^ Scale bars shown in TEM images correspond to 100 nm. (b) Schematic representation of the two‐stage mechanism proposed for the changes in copolymer morphology that are observed during thermal annealing of a binary mixture of 21±2 nm and 48±5 nm diblock copolymer spheres at 150 °C. The *n*, *m* and *p* values refer to the number density of each type of nanoparticle. In Stage 1, the smaller PLMA_39_‐PBzMA_97_ spheres undergo partial dissociation to form copolymer chains, which then become incorporated into the larger spheres to produce hybrid spheres with a mean diameter greater than 48 nm. If the volume fraction of these smaller spheres is less than 0.20, this is the final copolymer morphology. However, using higher volume fractions of this component leads to Stage 2, whereby the 21 nm spheres undergo fusion with the larger hybrid spheres to form weakly anisotropic transient species. The latter subsequently undergo fission—most likely mediated by incorporation of further PLMA_39_‐PBzMA_97_ chains—to form spheres of intermediate size (e.g., 36 nm diameter). Figure adapted from Ref. [44] with permission.

Cornel et al. also undertook a fundamental study to gain a better understanding of the behaviour of PLMA_39_‐PBzMA_x_ spherical nanoparticles during thermal annealing.^[44]^ More specifically, in situ SAXS studies revealed that heating a 1 % w/w dispersion comprising a binary mixture of relatively small PLMA_39_‐PBzMA_97_ spheres (core diameter=21±2 nm) and relatively large PLMA_39_‐PBzMA_294_ spheres (core diameter=48±5 nm) in *n*‐dodecane up to 150 °C led to the formation of spherical nanoparticles of intermediate size (core diameter=36±4 nm) on cooling to 25 °C, see Figure 21a. This scattering experiment was supported by TEM analysis (see Figure 21a). However, when the two types of initial nanoparticles were separately exposed to the same thermal annealing conditions, no size change occurred. For the smaller nanoparticles, solvation of the PBzMA cores owing to ingress by hot solvent occurred at 150 °C. In contrast, no solvation of the larger nanoparticles was observed. Furthermore, thermal annealing of the smaller nanoparticles at 150 °C resulted in a significant reduction in their mean aggregation number. A two‐stage mechanism was proposed to account for the formation of the intermediate‐sized spheres (see Figure 21b). During the first stage, the smaller PLMA_39_‐PBzMA_97_ nanoparticles undergo partial dissociation and the resulting free copolymer chains then become incorporated into the larger PLMA_39_‐PBzMA_294_ nanoparticles, which leads to an increase in their size. The second stage involves fusion of the remaining smaller spheres with the new ‘hybrid’ spheres, which results in the formation of weakly anisotropic transient species that subsequently undergo fission—most likely because of further incorporation of individual PLMA_39_‐PBzMA_97_ chains—to produce spheres of intermediate size.^[44]^

Direct experimental evidence for the rapid exchange of individual copolymer chains between sterically‐stabilized spherical nanoparticles at elevated temperature was obtained for a PLMA‐PMMA PISA formulation in *n*‐dodecane.^[46]^ Time‐resolved small‐angle neutron scattering (TR‐SANS) was used to analyse a binary mixture of fully hydrogenous PLMA_39_‐PMMA_55_ and core‐deuterated PLMA_39_‐d_8_PMMA_57_ spherical nanoparticles (each with a mean core diameter of ∼20 nm) after heating at 150 °C for just 3 min. The TR‐SANS data revealed that hybrid spheres with mixed cores (i.e., comprising both the PMMA_55_ and d_8_PMMA_57_ blocks) were obtained after this annealing protocol.

Moreover, a similar mixture of PLMA_39_‐PMMA_94_ and PLMA_39_‐d_8_PMMA_96_ required a longer annealing time (8 min) to produce spheres with mixed cores, which suggests that the rate of copolymer exchange depends on the DP of the core‐forming block. Furthermore, relatively slow copolymer exchange was observed even at 80 °C, which is below the *T*_g_ of the core‐forming PMMA block.^[46]^ These findings are consistent with previous TR‐SANS studies performed by Lund et al. and Bates, Lodge and co‐workers on block copolymer nanoparticles prepared by traditional post‐polymerization processing, rather than PISA.[^46^, ^98^, ^99^, ^100^, ^101^]

### 2.9 Potential Applications

Given the relative ease of production of these sterically‐stabilized nanoparticles and the proven scalability of RAFT polymerization chemistry,^[102]^ some of the above PISA formulations offer potential industrial applications. For example, Charleux et al. suggested that the film‐forming nature of their P(2‐EHA)‐PMA spherical nanoparticles prepared in *iso*‐dodecane[^33^, ^35^, ^79^] may be useful for certain cosmetics formulations (e.g., nail varnish). This particular PISA formulation was developed in collaboration with L'Oréal.^[34]^

The vesicle‐to‐worm transition observed by Derry et al. when heating PSMA‐PBzMA vesicles in mineral oil up to 150 °C resulted in a significant increase in both *G′* and complex viscosity. These observations led the authors to propose that this system might offer an interesting new high‐temperature oil‐thickening mechanism.^[75]^ However, the final *G′* value was relatively low (∼10^0^ Pa) and such worm gels exhibit strongly shear‐thinning behaviour.^[84]^ Thus, with the benefit of hindsight, this is probably not a realistic application unless (i) stronger worm gels can be designed and (ii) zero‐shear applications are identified. Indeed, the subsequent observation of a worm‐to‐sphere transition at higher temperature by Dorsman et al.^[76]^ casts further doubt on the prospect of a commercially exploitable oil‐thickening mechanism because the enhanced viscosity associated with the formation of worms is only observed over a relatively narrow temperature range.

Rymaruk and co‐workers demonstrated that PDMS‐PDMA worms could be employed as efficient viscosity modifiers (thickeners) for either *n*‐dodecane or low‐viscosity silicone oils.^[49]^ In this case, the reversible worm‐to‐sphere transition that occurred on mild heating could confer a useful processing advantage because it enables the thickening effect to be ‘switched off’ when desired. Moreover, core‐crosslinking such PDMS‐PDMA worms significantly lowers their critical gelation concentration, which leads to stronger thickening performance over a wide temperature range. On the other hand, such chemical derivatization eliminates their thermoresponsive behaviour, which may create processing problems.^[50]^

According to Zheng et al., diblock copolymer nanoparticles are potentially useful lubricant additives for automotive engine oils.^[23]^ In collaboration with Afton Chemicals, this Canadian team employed ATRP to prepare all‐acrylic block copolymers in THF. Subsequent transfer into non‐polar media via post‐polymerization processing led to the formation of sterically‐stabilized nanoparticles, which proved to be highly effective lubricants in the boundary lubrication regime.[^4^, ^23^] However, their laborious synthetic protocol—which also involved protecting group chemistry and photocrosslinking of the nanoparticle cores—is simply not commercially viable.

**Figure 22:**
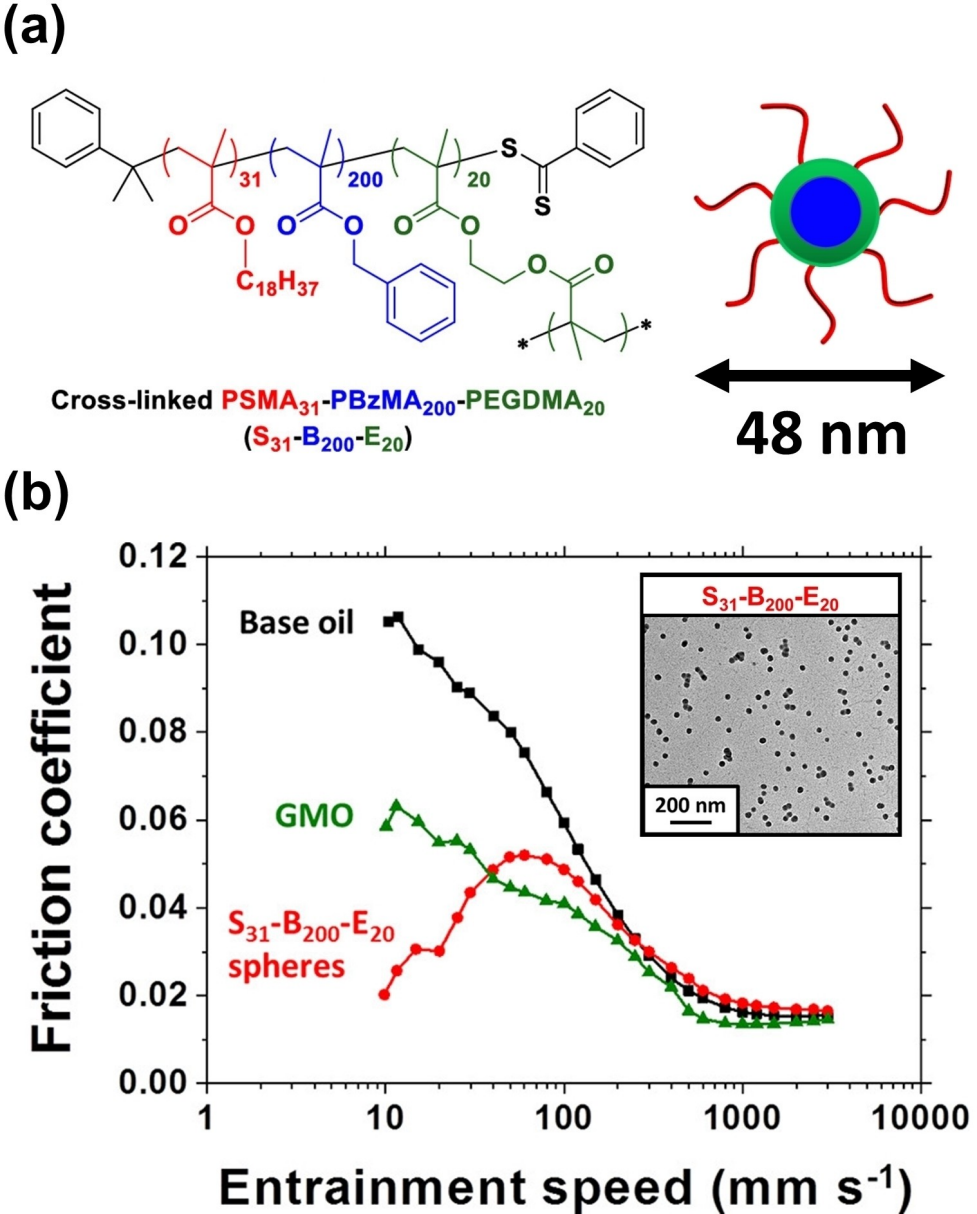
(a) Chemical structure and schematic representation of core‐crosslinked S_31_‐B_200_‐E_20_ triblock copolymer spherical nanoparticles. (b) Stribeck curves showing the change in friction coefficient with entrainment speed for a lubricating base oil alone (black squares), for 0.5 % w/w glyceryl monooleate (GMO, green triangles) in the same base oil, and for a 0.5 % w/w dispersion of 48 nm diameter PSMA_31_‐PBzMA_200_‐PEGDMA_20_ spheres dispersed in the same base oil (red circles). Data were recorded at a 20 % slide‐to‐roll ratio (SRR) under an applied load of 35 N at 100 °C. Figure adapted from Ref. [32] with permission.

A much more attractive route was developed by Derry et al., who prepared poly(stearyl methacrylate)‐poly‐(benzyl methacrylate)‐poly(ethylene glycol dimethacrylate) (PSMA_31_‐PBzMA_200_‐PEGDMA_20_) triblock copolymer nanoparticles of approximately 48 nm diameter in mineral oil via RAFT‐mediated PISA (see Figure 22a).^[32]^ Incorporating only 9 mol% EGDMA as the third block was sufficient to obtain durable core‐crosslinked spheres. Tribology experiments conducted using a mini‐traction machine (MTM; entrainment speed=10 to 3000 mm s^−1^; 20 % slide‐to‐roll ratio (SRR); applied load=35 N; 100 °C) confirmed that just 0.50 % w/w of such nanoparticles dramatically reduced the friction coefficient of an engine base oil within the boundary lubrication regime (entrainment speed=10–50 mm s^−1^) compared to a traditional friction modifier (glyceryl monooleate, GMO) employed at the same concentration (see Figure 22b). Given the undoubted scalability of the PISA formulation used to prepare such nanoparticles, such findings are expected to inform the design of next‐generation ultralow‐viscosity engine oil additives.

Working in collaboration with Lubrizol scientists, György et al. examined the relationship between enhanced nanoparticle adsorption and friction reduction.^[18]^ The adsorption of ∼27 nm epoxy‐functional P(LMA_50_‐*stat*‐GlyMA_9_)‐PMMA_67_, PLMA_63_‐PGlyMA_89_ and non‐functional PLMA_63_‐PMMA_67_ nanoparticles onto stainless steel from *n*‐dodecane was examined using a quartz crystal microbalance with dissipation (QCM‐D). Locating the epoxy groups within the stabilizer block of the nanoparticles led to a significantly higher adsorbed amount (7.6 mg m^−2^) compared to that obtained for epoxy‐core functional nanoparticles (3.7 mg m^−2^) or non‐functional nanoparticles (3.8 mg m^−2^) at 20 °C. Moreover, performing these QCM‐D adsorption experiments at 40 °C resulted in an even higher adsorbed amount (8.9 mg m^−2^) for the P(LMA_50_‐*stat*‐GlyMA_9_)‐PMMA_67_ nanoparticles, whereas a discernible *reduction* was observed for the other two types of nanoparticles (2.7 mg m^−2^ and 2.5 mg m^−2^ for the PLMA_63_‐PGlyMA_89_ and PLMA_63_‐PMMA_67_ nanoparticles, respectively). These observations suggest the P(LMA_50_‐*stat*‐GlyMA_9_)‐PMMA_67_ nanoparticles undergo *chemical adsorption* via ring‐opening of their epoxy groups by reaction with the Fe−OH groups on the surface of stainless steel, with a recent literature report supporting this hypothesis.^[103]^ In contrast, the other two type of nanoparticles merely undergo *physical adsorption*—most likely via hydrogen bonding interactions between the methacrylic ester groups on the PLMA chains and the Fe−OH groups.

**Figure 23:**
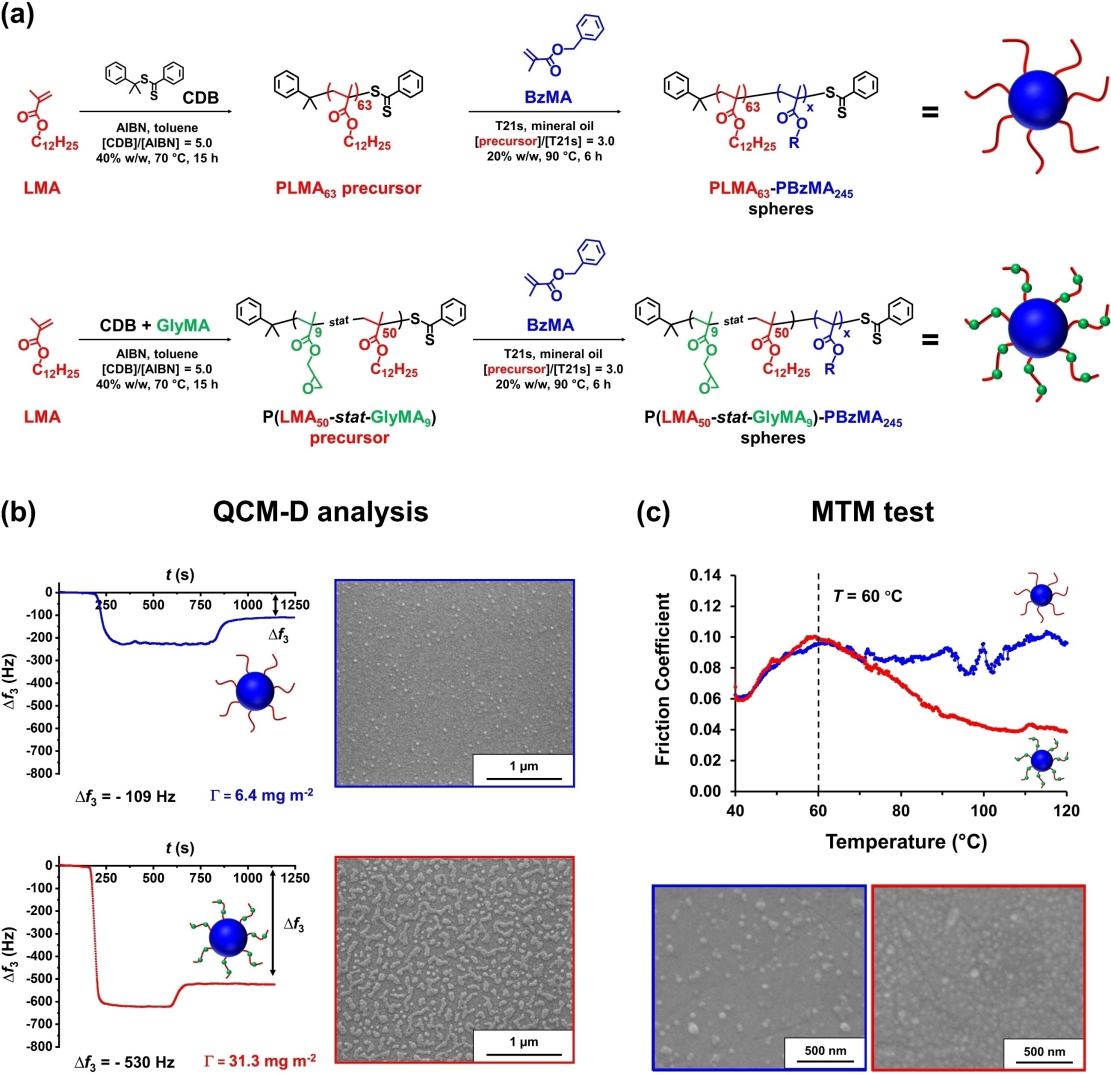
(a) Synthesis of PLMA_63_‐PBzMA_245_ and P(LMA_50_‐*stat*‐GlyMA_9_)‐PBzMA_245_ spherical nanoparticles via RAFT dispersion polymerization of BzMA in mineral oil at 20 % w/w solids. (b) QCM‐D data obtained for the adsorption of PLMA_63_‐PBzMA_245_ and P(LMA_50_‐*stat*‐GlyMA_9_)‐PBzMA_245_ nanoparticles in turn from 1.0 % w/w copolymer dispersions in n‐dodecane onto a stainless‐steel substrate at a flow rate of 0.50 mLmin^−1^ at 20 °C. Each curve is shown for a single measurement but good reproducibility was observed for duplicate experiments. The corresponding SEM images recorded for each nanoparticle‐coated stainless‐steel substrate after these QCM‐D experiments are also shown. The black double‐headed arrows indicate the final change in frequency (Δ*f*_3_). (c) Friction coefficient vs. temperature data obtained for a 2.5 % w/w dispersion of linear PLMA_63_‐PBzMA_245_ nanoparticles (blue data) and epoxy‐functional P(LMA_50_‐*stat*‐GlyMA_9_)‐PBzMA_245_ nanoparticles (red data). Data were recorded at an entrainment speed of 200 mms^−1^ with a slide‐to‐roll ratio (SRR) of 50 % under an applied load of 37 N. SEM images recorded for the MTM disks following these tribology experiments when using the PLMA_63_‐PBzMA_245_ nanoparticles (blue frame) and the P(LMA_50_‐*stat*‐GlyMA_9_)‐PBzMA_245_ nanoparticles (red frame), respectively. The epoxy groups in the latter nanoparticles clearly promote much stronger adsorption, which correlates with the significant reduction in friction coefficient observed above 60 °C. Figure adapted from Ref. [18] with permission.

Subsequently, the adsorption of ∼50 nm diameter P(LMA_50_‐*stat*‐GlyMA_9_)‐PBzMA_245_ and non‐functional PLMA_63_‐PBzMA_245_ nanoparticles onto stainless steel was compared at 20 °C using QCM‐D (see Figure 23a). These experiments indicated an adsorbed amount of 31.3 mg m^−2^ for the epoxy‐functional nanoparticles but only 6.4 mg m^−2^ for the non‐functional nanoparticles (see Figure 23b). Scanning electron microscopy was used to assess the corresponding fractional surface coverages, which were estimated to be 0.53 and 0.11 respectively. These two types of nanoparticles were subjected were then used for tribology studies. More specifically, MTM experiments were conducted at a constant entrainment speed of 200 mm s^−1^, an SRR of 50 % and an applied load of 37 N when heating from 40 °C to 120 °C. Above 60 °C—which corresponds to the onset of the boundary lubrication regime—the P(LMA_50_‐*stat*‐GlyMA_9_)‐PBzMA_245_ nanoparticles reduced the friction coefficient from 0.09 to 0.04 (see Figure 23c), whereas this parameter remained almost constant between 60 and 120 °C when using the non‐functional PLMA_63_‐PBzMA_245_ nanoparticles. SEM analysis of the worn stainless steel disks obtained after such MTM experiments indicated much higher surface coverage for the epoxy‐functional nanoparticles (see Figure 23c). Hence chemical adsorption of such nanoparticles can be directly related to the observed reduction in friction.

**Figure 24:**
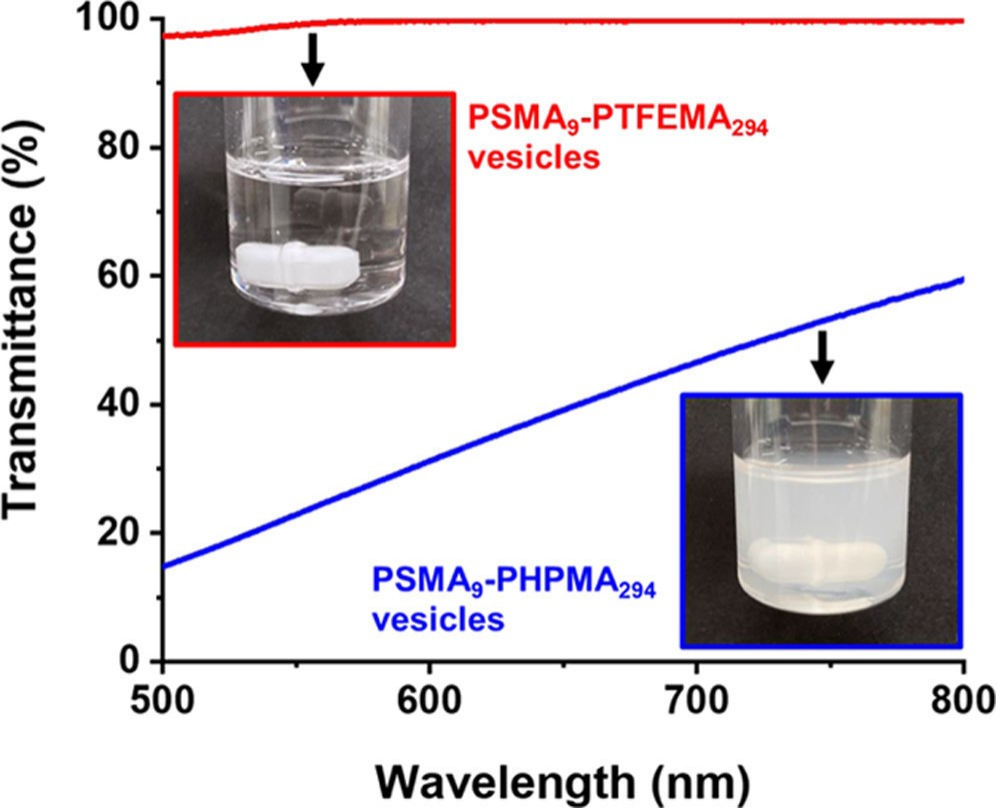
Transmittance vs. wavelength plots recorded at 25 °C for 0.50 % w/w dispersions of PSMA_9_‐PTFEMA_294_ (red data) (DLS diameter=237±24 nm) and PSMA_9_‐PHPMA_294_ (blue data) (DLS diameter=175±5 nm) vesicles in *n*‐dodecane. These vesicles were originally prepared at 25 % w/w in *n*‐dodecane by RAFT dispersion polymerization of either TFEMA or HPMA, respectively. Insets: digital photographs recorded for the same 0.50 % w/w dispersions at 25 °C to illustrate the marked difference in their physical appearance. Figure adapted from Ref. [96] with permission.

For some commercial applications, the high turbidity of vesicle dispersions is a potential drawback. Turbid dispersions (31 % transmittance at *λ*=600 nm) are typically observed even at a relatively low copolymer concentration, as illustrated for a 0.50 % w/w dispersion of PSMA_9_‐PHPMA_294_ vesicles in *n*‐dodecane (see Figure 24). The relatively large particle size of such vesicles (DLS diameter=175±5 nm) plus the refractive index difference between the vesicles and the continuous phase leads to strong light scattering. However, if there was no refractive index difference there would be minimal light scattering. Accordingly, György et al. matched the refractive index of the membrane‐forming PTFEMA block to that of the solvent to produce the first example of a highly transparent dispersion of diblock copolymer vesicles.^[96]^ More specifically, this approach enabled more than 99 % transmittance (at *λ*=600 nm) to be obtained for a 0.50 % w/w dispersion of PSMA_9_‐PTFEMA_294_ vesicles (DLS diameter=237±24 nm) in *n*‐dodecane at 25 °C (see Figure 24). Remarkably, 97 % transmittance was observed for the original 25 % w/w PSMA_9_‐PTFEMA_294_ vesicle dispersion at 20 °C. Moreover, similarly high transmittance could be achieved for the same PSMA_9_‐PTFEMA_294_ vesicles prepared at 25 % w/w solids in either *n*‐tetradecane at 50 °C or *n*‐hexadecane at 90 °C.

It is well‐known that block copolymer nanoparticles can be utilized as effective Pickering emulsifiers.[^104^, ^105^, ^106^, ^107^] In this context, using *hydrophobic* nanoparticles (readily prepared via PISA in non‐polar media) typically favors the formation of water‐in‐oil emulsions.[^65^, ^108^]

Interestingly, Cunningham et al. reported that. either water‐in‐oil (w/o) or oil‐in‐water (o/w) Pickering emulsions could be obtained when using 25 nm diameter PSMA_14_‐PNMEP_49_ nanoparticles depending on the shear rate employed for homogenization.^[65]^ More specifically, w/o emulsions were obtained when using a low shear rate (e.g., hand‐shaking) for homogenization, whereas only o/w emulsions were produced when employing higher shear using an IKA Ultra‐Turrax T‐18 homogenizer. This is because homogenization under high shear caused in situ inversion of the initial hydrophobic PSMA_14_‐PNMEP_49_ spheres to form hydrophilic PNMEP_49_‐PSMA_14_ spheres.

In 2020, Rymaruk et al. used RAFT‐mediated PISA to prepare 123 nm diameter PSiMA_19_‐PBzMA_200_ spherical nanoparticles directly in a low‐viscosity silicone oil (dimethicone 5 or DM5, which has a solution viscosity of only 5 cSt). These nanoparticles were subsequently evaluated as Pickering stabilizers for oil‐in‐oil (o/o) emulsions.^[109]^ The droplet phase comprised castor oil, sunflower oil or tall oil fatty acid (TOFA 26 %) within a continuous phase of DM5. Optical microscopy studies indicated that such Pickering emulsions were stable for at least two months when using a nanoparticle concentration of 2 % w/w. The PSiMA_19_ precursor was prepared using a carboxylic acid‐functional RAFT agent, which enabled convenient fluorescent labeling of the nanoparticles via esterification with a pyrene derivative. Thus fluorescence microscopy could be used to confirm nanoparticle adsorption at the surface of castor oil droplets. Furthermore, statistical copolymerization of LMA with BzMA when generating the core‐forming block produced PSiMA_19_‐P(BzMA_175_‐*stat*‐LMA_25_) nanoparticles, which enabled a much broader range of biosourced oils to be used for the preparation of o/o Pickering emulsions. However, this improvement was rather sensitive the LMA content of the core‐forming block: increasing the LMA content from 12.5 mol% to 18 mol% merely resulted in highly aggregated, unstable Pickering emulsions.

The first example of w/o Pickering nanoemulsions using diblock copolymer spheres obtained via PISA was reported by Hunter et al.,^[108]^ who used 28 nm PSMA_32_‐PTFEMA_53_ spherical nanoparticles prepared in *n*‐dodecane. Initially, addition of water to such hydrophobic nanoparticles followed by high‐shear homogenization led to the formation of a w/o Pickering macroemulsion. Subsequent high‐pressure microfluidization produced aqueous droplets of ∼600 nm diameter when using deionized water. However, addition of salt (NaCl) to the aqueous phase prior to emulsification resulted in the formation of much finer droplets. For example, DLS studies indicated that a mean aqueous droplet diameter of ∼250 nm was obtained in the presence of 0.11 M NaCl.^[108]^ Moreover, systematic variation of the nanoparticle concentration, applied pressure or the number of passes through the microfluidizer enabled the droplet diameter to be tuned.^[108]^ The long‐term stability of such nanoemulsions was assessed using analytical centrifugation. A substantial enhancement in stability was observed when employing higher NaCl concentrations (≥0.11 mol dm^−3^).^[108]^ In a related study, highly transparent w/o emulsions were prepared using refractive index‐matched PLMA_39_‐PTFEMA_800_ spherical nanoparticles prepared in *n*‐dodecane. Furthermore, combining these hydrophobic particles with hydrophilic poly(glycerol monomethacrylate)‐poly(2,2,2‐trifluoroethyl methacrylate) (PGMA_56_‐PTFEMA_500_) spherical nanoparticles enabled the rational design of an oil‐in‐water‐in‐oil (o/w/o) Pickering double emulsion that exhibited ∼90 % transmittance across the whole visible spectrum.^[110]^

## 3 Conclusions and Outlook

RAFT‐mediated PISA syntheses conducted in non‐polar media enable the rational design of a wide range of interesting new hydrophobic nanoparticles of tunable size, shape and chemical functionality. Such nano‐objects can be designed to adsorb efficiently at either solid or liquid interfaces, to be highly transparent, to form much stronger gels or to exhibit thermoresponsive behaviour.

In situ spectroscopic and scattering studies during PISA have provided vital new information, including polymerization kinetics, the evolution of particle size and morphology, verification of an ‘inward growth’ mechanism for vesicles and the rate of loss of the RAFT chain‐ends. They have also shed new light on the morphology transitions, micelle fusion/fission processes and the extent of copolymer chain exchange that can occur at elevated temperature.

However, if genuine commercial applications are to be realized for such functional diblock copolymer nanoparticles, considerable effort must be focused on minimizing the intrinsic color, malodor and additional cost associated with RAFT chemistry. For example, targeting a relatively high core‐forming block DP should mitigate such problems but would the production of relatively large nanoparticles be useful in a commercial context? Notwithstanding the proven scalability of RAFT polymerization, it may be sensible to switch to using either existing or future pseudo‐living radical polymerization chemistries for PISA syntheses in non‐polar media if its intrinsic disadvantages cannot be satisfactorily addressed. Nevertheless, it seems clear that RAFT‐mediated PISA has highlighted the broad potential of this field. Moreover, much of the knowledge gained should be applicable if the same (or similar) diblock copolymer nano‐objects are targeted using alternative synthetic protocols.

## Conflict of interest

The authors declare no conflict of interest.

4

## Biographical Information

*Csilla György obtained her BSc and MSc from Eötvös Loránd University (Hungary) and studied for her PhD degree in the Armes group at U. Sheffield. Her CDT PhD project was focused on preparing diblock copolymer nanoparticles in non‐polar media and was sponsored by EPSRC and Lubrizol Ltd. She received the 2023 Macro Group Jon Weaver prize for the best polymer science PhD thesis in the UK and is currently a postdoctoral researcher in Prof. B. Sumerlin‘s group at U. Florida*.

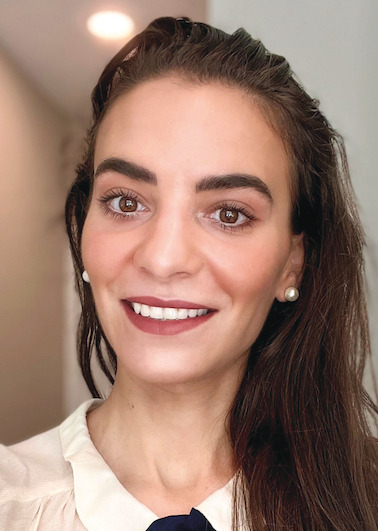

## Biographical Information

*Prof. Steve Armes FRS is the Firth Professor of Chemistry at the University of Sheffield. He has published 715 papers (>46,000 citations, H‐index*=*124), mainly in the field of polymer colloids. Over the past decade or so, he has focused on the development of polymerization‐induced self‐assembly (PISA) for the rational and efficient synthesis of a wide range of block copolymer nanoparticles*.

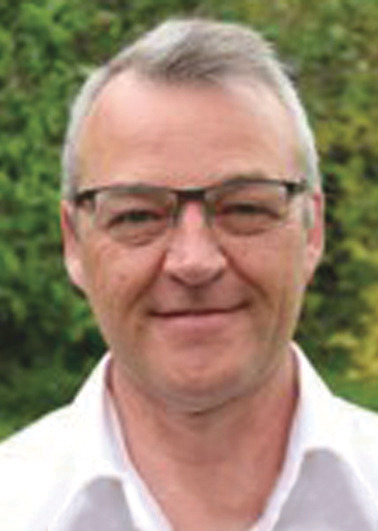

## Data Availability

Data sharing is not applicable to this article as no new data were created or analyzed in this study.
